# Dynamic changes of volatile substances and their driving factors during curing and fermentation of Hainan cigar

**DOI:** 10.1186/s40643-026-01088-3

**Published:** 2026-06-29

**Authors:** Wanyu Yue, Liang Zhao, Zhen Chen, Qinglin Guan, Yixiao Jin, Jun Wang, Juan Zhang, Zheng Peng

**Affiliations:** 1 Key Laboratory of Industrial Biotechnology, Ministry of Education, School of Biotechnology, Jiangnan University, 1800 Lihu Road, Wuxi, 214122 China; 2 Science Center for Future Foods, Jiangnan University, 1800 Lihu Road, Wuxi, 214122 China; 3 Technology Center, China Tobacco Zhejiang Industrial Co., Ltd. 118 Kehai Road, Xihu District, Hangzhou, 310024 China

**Keywords:** Cigar tobacco leaves, Air-curing, Fermentation, Microbial community, Volatile flavor compounds, Enzyme activity

## Abstract

Air-curing and fermentation are pivotal for cigar flavor, shaped by moisture dynamics, enzymatic activity, and microbial metabolism. This study comprehensively characterized Hainan cigar tobacco leaves (CTLs) across these processes, integrating volatile metabolomics, enzyme assays, high-throughput microbial sequencing, functional prediction, and correlation analyses to unravel the regulatory network of water activity, microbial succession, and metabolite transformation. Water activity served as the core environmental regulator. Low water activity in stem-drying selected stress-tolerant *Bacillus* and *Aspergillus*; rehydration to 0.75 in fermentation activated microbial metabolism, enriching functional specialists (*Pseudomonas* and *Aspergillus*). Volatile metabolites exhibited stage-specific dynamics: terpenoids peaked in final fermentation, the terpenoid content reached its peak during the final fermentation stage, characterized by a marked enrichment of key volatile aroma compounds, including neophytadiene, *β*-damascenone, and *β*-ionone. Functional analyses revealed upregulated amino acid degradation, lipid metabolism, and terpenoid transformation in fermentation. *Aspergillus* drove carotenoid/terpenoid precursor cleavage via oxidative enzymes to generate key C_13_-norisoprenoid aromas. *Pseudomonas* synergistically promoted high-impact aromas through secondary metabolism (e.g., polyketide biosynthesis). Other microbes cooperated in lipid oxidation, nitrogen-containing heterocycle formation, and aroma integration. This study distinguishes dehydration-driven air-curing and aroma-producing fermentation, identifies *Pseudomonas*, *Bacillus*, and *Aspergillus* as core functional drivers, and provides a theoretical basis for optimizing cigar tobacco production and precision quality control.

**Graphical abstract:**

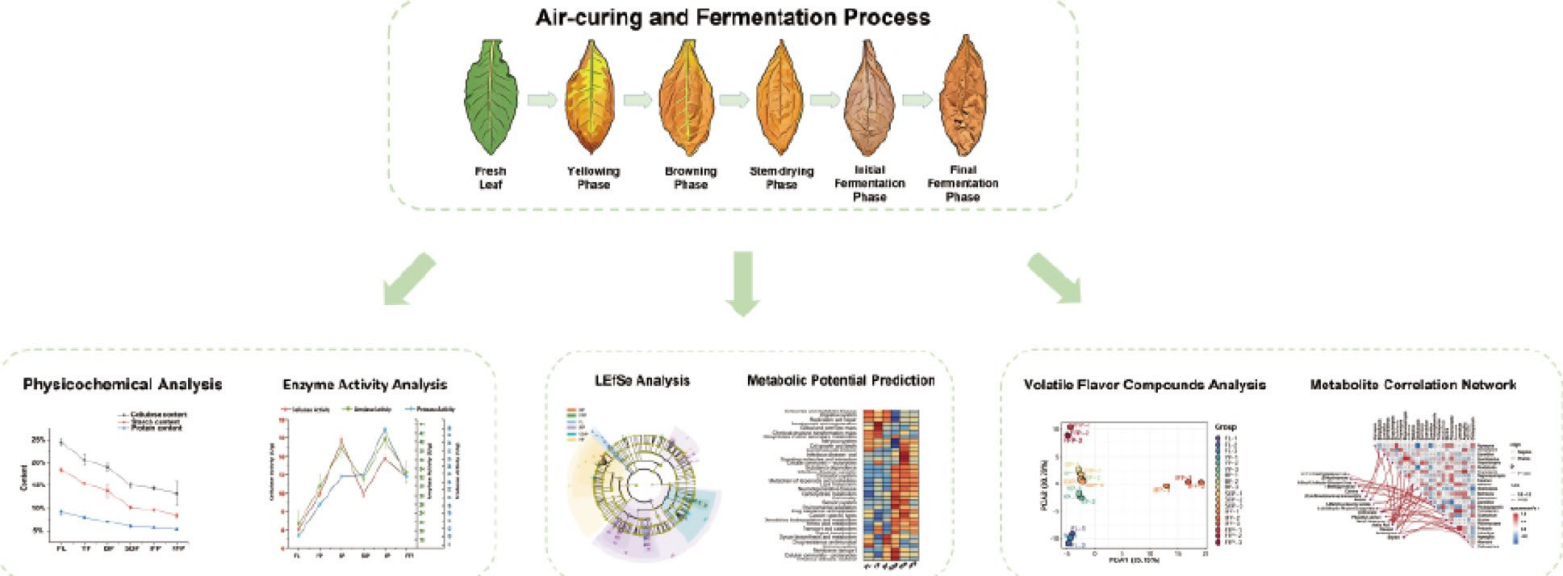

**Supplementary Information:**

The online version contains supplementary material available at 10.1186/s40643-026-01088-3.

## Introduction

Tobacco represents a significant economic crop worldwide (Chen et al. 2025). The development of cigar flavor quality fundamentally emerges from a systematic biochemical transformation process during the "curing-fermentation" phase, wherein moisture dynamics, enzymatic activities, and microbial metabolism interact synergistically to orchestrate continuous metabolic conversions (Zhang et al. 2023, 2025). In the initial phases of curing and fermentation, cellulose degradation promotes structural disintegration of cell walls, liberating entrapped aromatic amino acids and glycosidically bound flavor precursors (Tahir et al. 2025). Concurrently, the enzymatic conversion of starch and proteins into reducing sugars and free amino acids not only mitigates undesirable sensory characteristics—including harsh burnt notes and pungent irritants—but also establishes the essential biochemical substrates for subsequent flavor development through caramelization and Maillard reaction pathways (Banozic et al. 2020).

Cigar curing is a progressive air-drying–driven “starvation metabolism” process. Fundamentally, under precisely regulated temperature and humidity conditions, dehydration acts as the primary driving force that systematically shifts CTLs from active plant physiological metabolism toward a microbially dominated metabolic regime (Zhao et al. 2022). During this process, CTLs successively transition through the yellowing, browning, and stem-drying phases. Enzymatic browning and Maillard reactions jointly drive the visible transformation of leaf color from green to brown, while a concomitant directional succession of microbial communities occurs. Together, these processes synergistically promote the selective degradation of macromolecules such as cellulose, starch, and proteins, and facilitate the orderly accumulation of key flavor precursors. Structurally, the primary and secondary cell walls of CTLs are mainly composed of cellulose, hemicellulose, and pectin (Banozic et al. 2020). Studies have demonstrated that elevated cellulose content in tobacco can contribute to undesirable aroma characteristics, primarily owing to the formation of unpleasant sensory byproducts during cellulose pyrolysis. Volatile flavor compounds (VFCs) such as furfural and its derivatives detected in tobacco are largely derived from cellulose present in leaf tissues. Moreover, higher cellulose levels have been shown to be positively associated with increased formaldehyde formation, while concomitantly correlating with reduced levels of aromatic amines, collectively exerting a negative impact on overall flavor quality (Feng et al. 2021). The pyrolysis of tobacco cellulose produces several low‑molecular‑weight compounds, notably ketones and aldehydes. Starch, which accumulates as a major primary metabolite during tobacco growth, is progressively broken down during curing and fermentation. This degradation transforms starch into water‑soluble carbohydrates, thereby supplying key precursors for the biosynthesis of aroma‑active compounds (Gao et al. 2025a, b). Although starch serves as an important precursor for aroma formation, excessive residual starch in tobacco products can induce bitterness and flavor off-notes, and may also impair color development and aroma evolution during curing. Consequently, efficient starch degradation is a critical step in tobacco processing. Beyond reducing irritating constituents such as nicotine, the curing process optimizes the carbon–nitrogen balance of the leaf, thereby establishing a favorable substrate profile and reaction microenvironment for subsequent fermentation. In this context, curing represents an indispensable preparatory phase for the development of desirable cigar flavor quality (Banozic et al. 2020).

The aroma profile of cigars is highly complex and multifaceted, comprising a broad array of VFCs with distinct biochemical origins and structural characteristics. These compounds can be generally classified into several groups: floral and fruity aroma constituents derived from carotenoid degradation (e.g., *β*-damascenone and *β*-ionone); aldehydes and alcohols formed via lipid oxidation (e.g., nonanal, decanal, and hexanal); roasted and toasted notes generated through Maillard reactions, including pyrazines and furans; and aromatic compounds originating from the phenylalanine metabolic pathway, such as phenylacetaldehyde and phenethyl alcohol. In addition, phenolic compounds, terpenoids, and organic acids contribute synergistically to the modulation and stabilization of the overall aroma profile (Zhu et al. 2024; Zhang et al. 2025). The fermentation phase of cigar tobacco is a pivotal biochemical phase in flavor and quality development. Under controlled temperature and humidity conditions, functional microorganisms drive the degradation of macromolecules such as proteins and alkaloids, effectively reducing green off-odors and irritancy. Meanwhile, the sugars and amino acids released during this process act as essential precursors that, via Maillard reactions and Strecker degradation (Xing and Yaylayan 2024; Qi et al. 2025), generate a broad spectrum of key aroma compounds—including aldehydes, ketones, esters, and pyrazines—thereby underpinning the mellow mouthfeel and complex aromatic profile characteristic of cigars (Qin et al. 2021). Concurrently, the fermentation process substantially enhances the physical properties of CTLs, whereby their chromaticity, ductility, and combustion performance are precisely modulated to meet the stringent requirements of high-quality tobacco products (Zheng et al. 2022). Thus, fermentation essentially represents a flavor-forming stage that directionally transforms cured "raw materials" into finished tobacco products, and it exerts a determinative effect on shaping the ultimate sensory style and commercial value of cigars. Currently, research pertaining to the microbial communities of CTLs has primarily focused on the fermentation stage, with particular emphasis on the screening of functional microorganisms and the successional dynamics of microbial community structures (Si et al. 2023). The dynamic shifts in microbial communities and their metabolic functions across different tobacco leaf positions during the curing process remain largely elusive; moreover, systematic and sequential analyses that integrate the curing and fermentation stages are still relatively scarce. These knowledge gaps have, to a certain extent, impeded an in-depth understanding of the holistic mechanisms underlying cigar flavor formation (Li et al. 2024).

This study systematically explored the intricate interplay among moisture content, enzyme systems, microbial communities, and metabolites during these processes, aiming to delineate the key driving factors governing quality formation in CTLs. Sample collection encompassed four critical curing phases (fresh leaf phase, yellowing phase, browning phase, and stem-drying phase), as well as the early and final phases of fermentation. High-throughput sequencing technology was employed to characterize the structural composition of bacterial and fungal communities. Redundancy analysis revealed significant correlations between microbial assemblages, enzyme activities, and curing-associated environmental factors. Based on microbial annotation results, the functional gene potential of bacterial and fungal communities was predicted and deciphered using the KEGG and MetaCyc databases, respectively. From the perspective of metabolic pathways, this analysis elucidated the key biochemical processes potentially mediated by microorganisms across different phases. Collectively, these findings provide valuable insights into the functional microbe-driven metabolic transformation mechanisms of CTLs during curing and fermentation, and offer robust theoretical underpinnings for process optimization and quality modulation.

## Materials and methods

### Collection of the cigar tobacco samples

Cigar tobacco was cultivated under a standardized agronomic protocol that included basal fertilization, field sanitation, and prophylactic pest management prior to transplanting. During early vegetative growth, integrated water–nutrient supply and pest suppression were maintained through topdressing, selective pesticide application, and intertillage. In the mid-to-late developmental stages, canopy management—comprising lower leaf removal, apical meristem excision (topping), and axillary bud suppression (sucker control)—was implemented alongside optimized fertilization to promote uniform maturation and enhance leaf quality. Middle-stalk leaves were harvested in July 2024 from a production site in Hainan Province, China, mounted on bamboo poles, and subjected to natural air-curing for 45 days. Temperature and relative humidity during curing were dictated by the local climate and were continuously monitored and recorded. Specimens were collected at the fresh leaf stage (FL) and at three time points during air-curing: yellowing phase (YP, day 10), browning phase (BP, day 25), and stem-drying phase (SDP, day 45). Following curing, the leaves underwent artificial pile fermentation, with additional sampling at the initial fermentation phase (IFP, day 15) and final fermentation phase (FFP, day 45). During fermentation, the core temperature of the piled leaves reached approximately 42 °C, and the relative humidity was maintained at 80%. At each sampling point, approximately 20 g of interveinal lamina tissue was aseptically excised from a consistent leaf position using sterile instruments, immediately flash-frozen in liquid nitrogen, and stored at − 80 °C for subsequent DNA extraction and enzyme activity assays. Three independent biological replicates were prepared per time point to ensure analytical rigor and reproducibility.

### Determination of physicochemical properties of CTLs

For samples from the six phases of curing and fermentation, moisture content, water activity, contents of cellulose, starch, and protein, as well as activities of protease, amylase, and cellulase were determined. The moisture content of CTLs was measured by the oven-drying weight loss method. After shredding and homogenizing the samples, an accurately weighed aliquot was dried at 105 °C to a constant weight, and the moisture content was calculated based on the mass difference before and after drying. The water activity was detected using a LabSwift-aw water activity meter (Novasina, Switzerland).

Approximately 0.1 g of each sample was weighed. The contents of cellulose, starch, and protein were assayed using corresponding detection kits purchased from Beijing Solarbio Science & Technology Co., Ltd. Meanwhile, the activities of cellulase, amylase, and protease were determined with micro-enzyme-linked immunosorbent assay (ELISA) kits from the same company, strictly following the manufacturer’s protocols. All measurements were performed using a microplate reader, with six biological replicates set for each sample. Enzyme activities were calculated according to the formula specified in the kit instructions (Lee et al. 2021).

### HS–SPME–GC–MS analysis

VFCs in CTLs were analyzed by headspace solid-phase microextraction (HS-SPME) coupled with gas chromatography–mass spectrometry (GC–MS). Briefly, leaf samples were dried at 40 °C, ground into a fine powder, and 1.5 g of each was placed into a headspace vial with ethyl decanoate (0.2155 g/L) added as an internal standard. Volatiles were extracted using a 50/30 μm DVB/CAR/PDMS fiber (Supelco, USA) at 60 °C for 30 min under headspace conditions. GC–MS analysis was carried out on an Agilent 7890A/5975C system equipped with a DB-5MS capillary column (30 m × 250 μm × 0.25 μm). The SPME fiber was desorbed at 250 °C for 5 min in splitless mode. Helium served as the carrier gas at 1 mL/min, and the oven temperature was programmed from 40 °C (held 2 min) to 250 °C at 10 °C/min, followed by a 6-min hold. Mass spectra were acquired in full-scan mode (m/z 33–400) at 70 eV, with ion source and transfer line temperatures set at 210 °C and 280 °C, respectively, and a solvent delay of 3 min. Raw data were processed using ChromaTOF 4.3X software for peak detection, alignment, deconvolution, and integration. Compound identification was based on spectral matching against the WILEY 8.0 and NIST 14 databases, and relative quantification was performed via the internal standard method using 2-octanol (0.2155 g/L), with results expressed as the ratio of analyte peak area to that of the internal standard. The quantification of VFCs was performed utilizing a semi-quantitative approach that relies on peak areas, with ethyl decanoate serving as the internal standard at a known concentration.

### DNA extraction and amplicon sequencing of microbial communities

CTLs (5 g) were added to a sterile conical flask containing 50 mL of sterile saline (0.85% NaCl, w/v). The mixture was homogenized by shaking at 2500 rpm for 30 min. After repeated rinsing of the leaves with sterile saline, the resulting eluate was filtered and centrifuged at 7000 × g for 15 min at 4 °C. The supernatant was discarded, and the microbial pellet was collected. Total DNA was extracted using the MagPure Soil DNA LQ Kit (Magan). DNA purity and concentration were evaluated by agarose gel electrophoresis. All samples were processed in triplicate to ensure biological reproducibility, and the extracted DNA was stored at − 20 °C until further sequencing and analysis.

The extracted DNA served as the template for PCR amplification. The V4–V5 hypervariable regions of the bacterial and archaeal 16S rRNA gene were amplified with primers 515F (5′-GTGCCAGCMGCCGCGGTAA-3′) and 907R (5′-CCGTCAATTCMTTTRAGTTT-3′) (Yang et al. 2022). For fungal communities, the ITS1 region was amplified using primers ITS1F (5′-CTTGGTCATTTAGAGGAAGTAA-3′) and ITS2R (5′-GCTGCGTTCTTCATCGATGC-3′) (Porter and Golding 2012). PCR reactions were performed in a 25 μL system containing 5 μL Q5 Reaction Buffer (5 ×), 5 μL Q5 High‑Fidelity GC Buffer (5 ×), 0.25 μL Q5 High‑Fidelity DNA Polymerase (5 U/μL), 2 μL dNTPs (2.5 mM), 1 μL of each primer (10 μM), and 2 μL DNA template, with nuclease‑free water added to a final volume of 25 μL. Following verification of amplicon size by agarose gel electrophoresis, PCR products were subjected to Illumina‑based high‑throughput sequencing for subsequent analysis.

### Statistical analysis

Statistical analysis was performed using SPSS (version 25.0.0.2), where one-way ANOVA was applied to assess the physicochemical properties and enzyme activities. Data are expressed as mean ± standard deviation and were graphically visualized using GraphPad Prism (version 9.3.0).Venn diagrams, stacked bar charts, box plots, and *β*-diversity principal coordinate analysis (PCoA) plots were generated using the GenesCloud platform (https://www.genescloud.cn). Partial Least Squares-Discriminant Analysis (PLS-DA) was conducted in SIMCA 14.1 to identify differentially abundant metabolites across cigar samples. The model was constructed using a random sampling strategy, and its fit was validated through 200 permutation tests. Potential overfitting was assessed by examining the regression lines and intercepts of R^2^ and Q^2^. Variables with Variable Importance in Projection (VIP) scores ≥ 1 were considered key discriminative features.The distributions of differential VFCs were visualized as bar charts created in Origin 2024b. Metabolic heatmaps were generated in RStudio (version 4.2.2) using the "pheatmap" package.Linear discriminant analysis effect size (LEfSe) was applied to identify fungal and bacterial taxa exhibiting significant differences across groups. Using a threshold of *α* = 0.05, the factorial Kruskal–Wallis rank-sum test was first performed to select taxa with significant abundance differences, followed by effect size estimation using logarithmic linear discriminant analysis (LDA) scores. Taxa with an LDA score > 4 were retained for further analysis.

Sequences sharing 100% identity were clustered into amplicon sequence variants (ASVs). Taxonomic annotation of bacterial and fungal ASVs was performed by aligning 16S rRNA gene sequences against the SILVA database (release 138) and ITS1 sequences against the UNITE database (release 8.0), respectively. To account for uneven sequencing depth, all samples were rarefied to 95% of the minimum sequence count per sample.Microbial community functional potential was predicted using PICRUSt2 (Phylogenetic Investigation of Communities by Reconstruction of Unobserved States; version 2.5.2).Kendall's correlation analysis between enzyme activity, water activity, and VFCs was performed using the psych package in R (v4.2.2). Correlations with *p* < 0.05 and |r|> 0.8 were considered statistically significant. Spearman's correlation analysis was also conducted to examine associations between microbial taxa and characteristic flavor components, with significance defined as *p* < 0.05 and |r|> 0.6. Correlation network heatmaps were visualized using OmicStudio tools (https://www.omicstudio.cn/tool).

## Results

### Physicochemical changes of CTLs

**Fig. 1:**
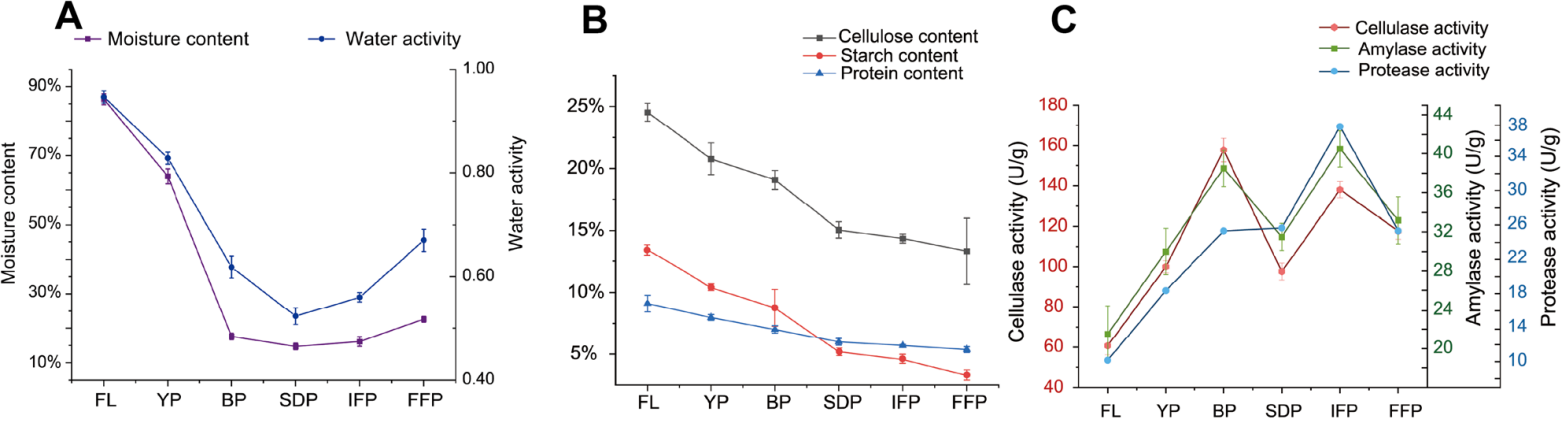
Dynamics of moisture, macromolecules, and enzyme activities during curing and fermentation of CTLs. **A** Temporal profiles of moisture content and water activity. **B** Temporal profiles of cellulose, starch, and protein contents. **C** Temporal profiles of cellulase, amylase, and protease activities. FL, Fresh leaf; YP, Yellowing phase; BP, Browning phase; SDP, Stem-drying phase; IFP, Initial fermentation phase; FFP, final fermentation phase

As illustrated in Fig. 1, the moisture content of CTLs decreased continuously from 86% at the initial phase with the advancement of the curing process, and finally stabilized at 23% during the fermentation stage. Throughout the curing period, both the moisture content and water activity of CTLs exhibited a steady declining trend. From the fresh leaf phase to the YP, the moisture content dropped markedly (to 61% and 0.67, respectively), which indicates the onset of continuous dehydration in CTLs. The most significant reduction in moisture content occurred between the YP and the BP. By the SDP, both the moisture content (14%–18%) and water activity (0.50–0.54) of the CTLs reached their minimum values. Notably, a water activity below 0.60 serves as the critical threshold to inhibit the growth of most microorganisms and maintain the biochemical stability of CTLs. During fermentation, the moisture content and water activity of the CTLs increased slightly by regulating the ambient temperature and humidity.

From the fresh leaf phase to the BP, the activities of cellulase, amylase, and protease kept rising and peaked at the BP. From the BP to the SDP, cellulase and amylase activities decreased, whereas protease activity remained largely unchanged. In the early fermentation phase, the activities of cellulase, amylase, and protease increased again, followed by a gradual decrease at the FFP.

### Metabolomic changes of CTLs

**Fig. 2:**
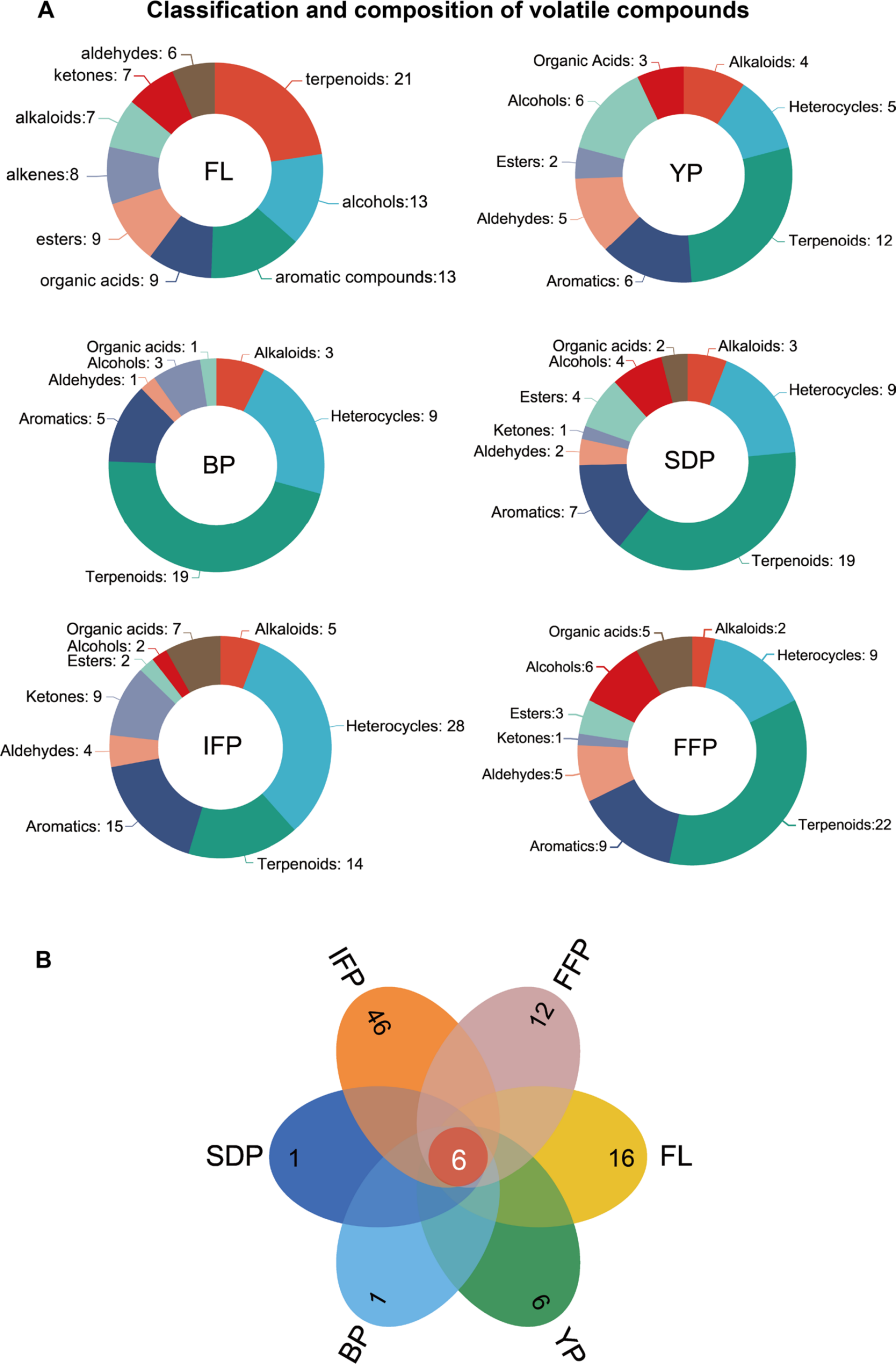
Analysis of volatile compound profiles across different stages of cigar processing. **A** Classification and composition of volatile compounds in cigar tobacco at different processing stages. Embedded values indicate compound counts. **B** Venn diagrams of volatile compound types across different stages of cigar processing

During the processing of CTLs, the composition and content of VFCs undergo significant dynamic changes— a phenomenon that directly mirrors the continuous transformation of internal aroma precursors and the gradual reconstruction of the overall flavor system. VFCs in cigar tobacco leaves were detected and screened by HS‑SPME-GC‑MS. In total, 157 volatile compounds were identified, with the corresponding data listed in Supplementary Table 1. In the early IFP, CTLs contain the largest number of volatile compounds, while fresh cigar leaves have the fewest (as shown in Supplementary Fig. 1). In terms of the variation in types of volatile components, the number of terpenoid compounds in CTLs increased progressively during the curing phase, whereas the types of ester compounds decreased notably, indicating that the curing process promotes the transformation of carotenoids and terpenoid precursors (as shown in Fig. 2). Upon entering the early fermentation phase, the diversity of heterocyclic compounds increased remarkably.

Regarding changes in the content of volatile substances, a distinct phasic regularity was observed. Among these compounds, terpenoids— as the core aroma components of cigars— accumulated continuously during the curing process, further increased significantly in the fermentation phase, and attained their peak level at the FFP. This finding demonstrates that terpenoids play a dominant role in the formation, enhancement, and maturation of the characteristic aroma of cigars, acting as a crucial material foundation for constructing the complex aroma profile of cigars. Overall, the number of volatile compound types exhibited an upward trend during the curing phase, with the highest diversity detected in the early fermentation phase.

The alkaloid content in cigar leaves decreases markedly during air curing, whereas terpenoids accumulate throughout fermentation (as shown in Supplementary Fig. 2). Alkaloids in CTLs displayed distinct phasic attenuation and transformation features during air-curing and fermentation. Nicotine content was the highest at the fresh leaf phase, far surpassing that of other alkaloids, and it constitutes a major source of the irritancy associated with cigars. Upon entering the YP, nicotine content dropped rapidly by over 60%, indicating that the early curing stage is pivotal for the rapid degradation and transformation of nicotine. Some nicotine derivatives appeared transiently during the curing phase: for example, 3-(4,5-dihydro-1H-pyrrol-2-yl) pyridine increased slightly in the YP, while cotinine reached a relatively high level in the same phase. This implies that nicotine undergoes oxidation, demethylation, and other reactions during curing to generate intermediate metabolites.

**Fig. 3:**
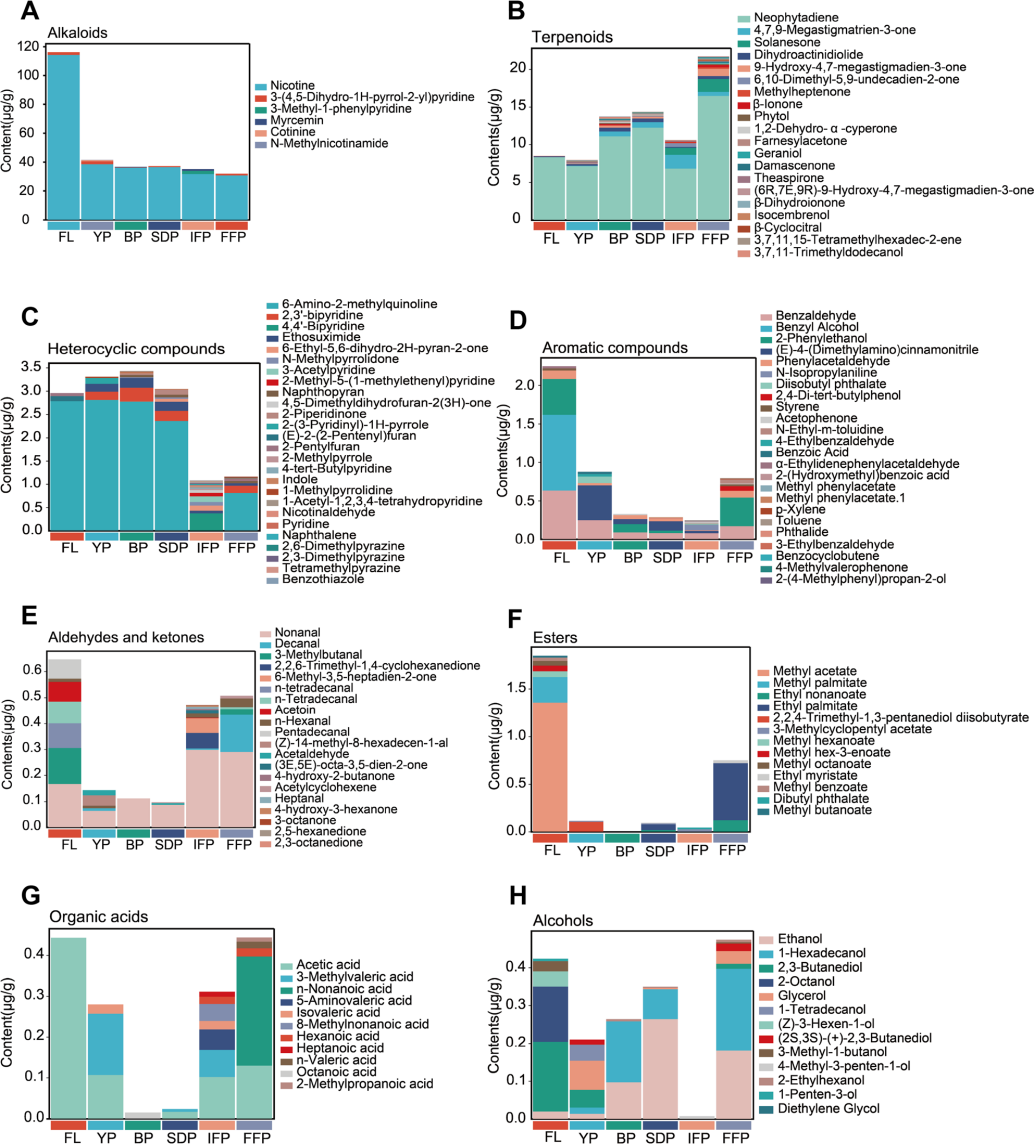
Dynamics of volatile compound concentrations during air-curing and fermentation of CTLs. **A** Alkaloids. **B** Terpenoids. **C** Heterocyclic compounds. **D** Aromatic compounds. **E** Aldehydes and ketones. **F** Esters. **G** Organic acids. **H** Alcohols

The air-curing stage is a critical period for the activation and cleavage of terpenoid precursors. As shown in Fig. 3, neophytadiene was the dominant terpenoid in fresh CTLs. Upon entering the BP, typical carotenoid cleavage products such as dihydroactinidiolide, *β*‑dihydroionone, 1,2‑dehydro‑*α*‑cyperone, and phytol began to appear and gradually increased, while the content of neophytadiene also rose significantly. During the IFP, the levels of some terpenoids (e.g., neophytadiene and dihydroactinidiolide) temporarily decreased, suggesting their potential role as substrates for further transformation. Meanwhile, compounds such as solanone, theaspirane, *β*‑ionone, and various hydroxy‑megastigmatrienones began to emerge or increase markedly, indicating that microbial and enzymatic activities collaboratively drive deep structural rearrangement of terpenoids in this phase.

By the FFP, both the diversity and total abundance of terpenoids reached their highest levels. Neophytadiene content increased significantly, accompanied by a notable rise in multiple terpenoids exhibiting floral, fruity, and woody characteristics, including solanone, *β*‑ionone, damascenone, theaspirane, *β*‑cyclocitral, valencene, cembrene, and eremophilene.

The content of heterocyclic compounds increased slightly during the curing phase and then declined markedly in the fermentation phase, presenting a "first rise and then fall" pattern. This indicates that as flavor precursors, heterocyclic compounds are further converted into substances with more complex structures and milder aroma profiles during fermentation. Meanwhile, after a significant reduction in the curing phase, the contents of esters, organic acids, aldehydes, and ketones rebounded notably in the fermentation phase, demonstrating that the fermentation process is a crucial stage for the regeneration and enrichment of these key aroma compounds.

Ester compounds displayed a variation pattern of "significant reduction during curing—subsequent regeneration and accumulation during fermentation" throughout the processing of CTLs. Upon entering the curing phase, the contents of most organic acids declined markedly, even dropping below the detectable limit—an observation potentially attributed to respiratory metabolic consumption, volatile loss, and conversion into other precursor substances. As fermentation progressed, particularly in the middle and late stages, certain short-chain and branched-chain fatty acids (e.g., n-nonanoic acid, hexanoic acid, heptanoic acid, n-valeric acid) gradually formed and accumulated (Sun et al. 2011), ethyl phenylacetate, an aromatic ester with floral and fruity aroma notes, is notably enriched during the SDP. This demonstrates that the esterification reaction between aromatic precursors and alcohol/acid substrates is further enhanced, which contributes to improving the aroma softness and complexity of CTLs (Long et al. 2020; Li et al. 2025).

During the SDP, the metabolic characteristics of CTLs are characterized by the in-depth transformation of nitrogen-containing compounds and the prominent accumulation of aromatic esters. In this phase, the content of 2-piperidone is notably higher than that in other phases, indicating that nitrogen-containing compounds (e.g., amino acids and alkaloids) undergo continuous decomposition and rearrangement to produce more lactam compounds—reflecting the profound transformation of proteins and nitrogen-containing precursors.

**Fig. 4:**
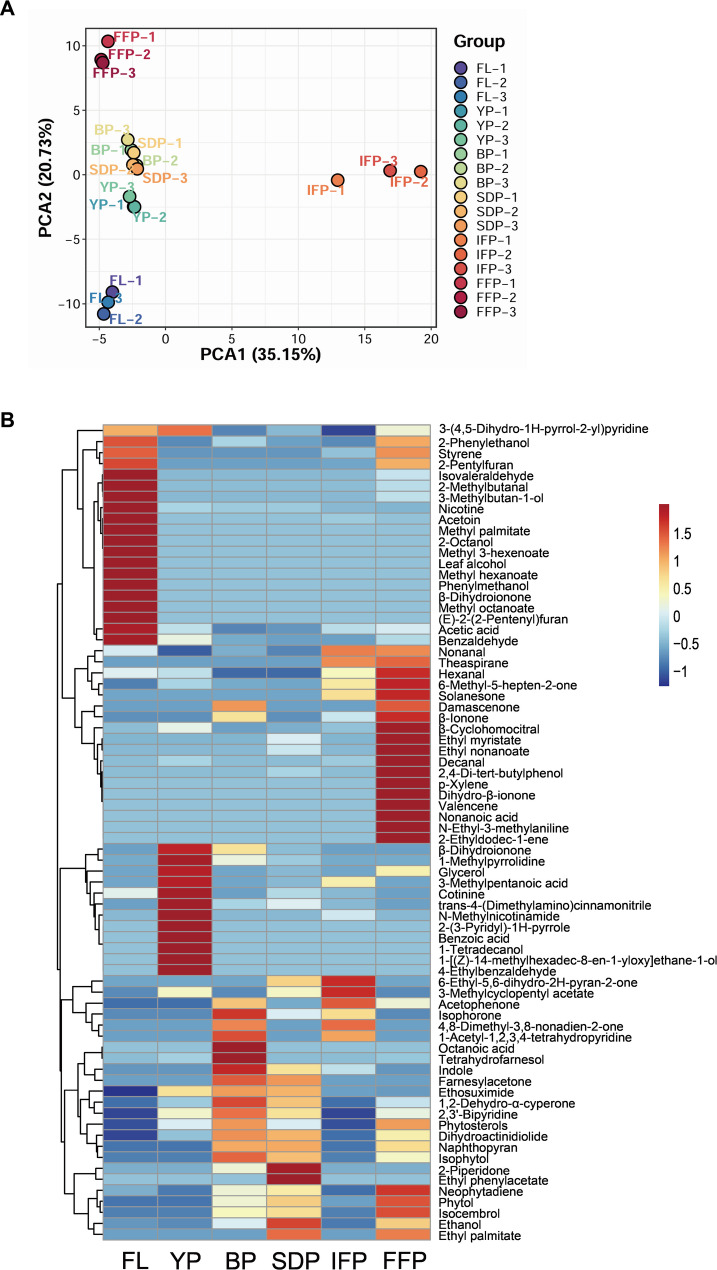
Analysis of characteristic VFCs at different phases of cigar processing. **A** Principal component analysis (PCA) score plots were used to analyze the characteristics of VFCs at different phases. **B** Heatmap of VFCs with VIP values greater than 1

To explore the dynamic evolution of metabolites during cigar tobacco processing, principal component analysis (PCA) was applied to the VFCs of all samples. The PCA score plot showed clear phase-specific clustering among samples from different phases (as shown in Fig. 4). The first two principal components together explained 55.88% of total variance, suggesting that metabolite profiles effectively distinguished processing phases and reflected underlying biochemical transformations. Based on variable importance in projection scores derived from the partial least squares-discriminant analysis model (as shown in Supplementary Fig. 3), initially identified differential metabolites were further examined to investigate their dynamic changes over different time periods during tobacco curing.

In fresh leaves, metabolites arising from lipid and amino acid degradation predominated, with several characteristic compounds showing distinct enrichment. Specifically, small‑molecule aldehydes, alcohols, and organic acids—including isovaleraldehyde, 2‑methylbutanal, 3‑methylbutan‑1‑ol, nicotine, acetoin, and acetic acid—were present at significantly higher levels compared to other stages, reflecting active tissue metabolism, vigorous amino acid turnover, and pronounced respiratory activity. Fatty acid methyl esters such as methyl palmitate, methyl hexanoate, methyl 3‑hexenoate, and methyl octanoate, along with lipid‑ and carotenoid‑derived compounds like leaf alcohol, 2‑octanol, phenylmethanol, and *β*‑dihydroionone, were also notably enriched at this stage. Upon transition to the YP, the metabolic profile shifted toward enhanced secondary metabolism. A marked increase in *β*‑dihydroionone indicated sustained carotenoid cleavage, providing key precursors for later aroma development. Concurrently, nitrogen‑containing heterocycles and nicotine derivatives—such as 1‑methylpyrrolidine, 2‑(3‑pyridyl)-1H‑pyrrole, cotinine, and N‑methylnicotinamide—accumulated significantly, pointing to active nicotine oxidation and nitrogenous compound metabolism. Overall, this phase was defined by the transformation of nitrogen‑containing species, ongoing lipid degradation, and the buildup of aromatic precursors, marking a metabolic shift from primary pathways toward aroma‑compound formation. During the BP, metabolism became further concentrated on terpenoids and fatty acid derivatives. The content of octanoic acid was significantly elevated relative to other stages, suggesting intensified fatty acid breakdown and oxidation that yielded abundant medium‑chain fatty acids as crucial substrates for subsequent esterification and flavor generation. At the same time, significant enrichment of tetrahydrofarnesol, a sesquiterpenoid alcohol, underscored active isoprenoid metabolism and terpenoid transformation in this period.

During the SDP, metabolic activity shifts toward the deep transformation of nitrogen‑containing compounds and the generation of aromatic esters. The metabolic profile at this stage is defined by a pronounced conversion of nitrogenous precursors and a marked accumulation of aromatic esters. Specifically, the 2-piperidone content in the SDP was more than twofold greater than that in the BP, indicating that nitrogen‑containing compounds—such as amino acids and alkaloids—undergo continuous decomposition and rearrangement to yield more lactam structures. This reflects the extensive breakdown and remodeling of proteins and other nitrogen‑rich precursors. Concurrently, ethyl phenylacetate, an aromatic ester with distinct floral‑fruity notes, was notably enriched during this phase. This enrichment suggests that esterification between aromatic precursors and alcohol/acid substrates is further enhanced, contributing to greater aroma softness and complexity in the CTLs (Long et al. 2020; Li et al. 2025).

The IFP is characterized by the highest metabolite diversity and the most vigorous transformation activity. On the one hand, nicotine and simple alkaloids are further diminished; on the other hand, nitrogen-containing heterocycles, ketones, aldehydes, and specific aromatic compounds are prominently enriched—encompassing pyridine, pyrazine, pyrrole derivatives, and various low-abundance flavor-active substances. This reflects the synergistic effects of microbial metabolism, amino acid degradation, and pre-Maillard reactions. The FFP, in contrast, is defined by the prominent enrichment of terpenoids and their oxidation/cleavage products as the core metabolic trait. Quantitatively, the total terpenoid concentration in the FFP was approximately 2.5-fold higher than that in fresh leaves, representing the most pronounced enrichment among all volatile classes detected. Among these compounds, the contents of neophytadiene, solanone, ionone, and a series of degraded or rearranged terpenoid-derived ketones attain peak levels throughout the entire process. This demonstrates that precursor substances such as carotenoids and chlorophyll side chains have been profoundly converted into key aroma components with typical cigar characteristics via continuous oxidation, cleavage, and reconstruction (Qin et al. 2021). Meanwhile, certain organic acids and ester compounds re-emerge and accumulate gradually during this phase, reflecting the ongoing oxidation, esterification, and slow secondary reactions in the fermentation system (Rahayu et al. 2017).

### Effects of moisture content, water activity, and three enzyme activities on the metabolome of CTLs

**Fig. 5:**
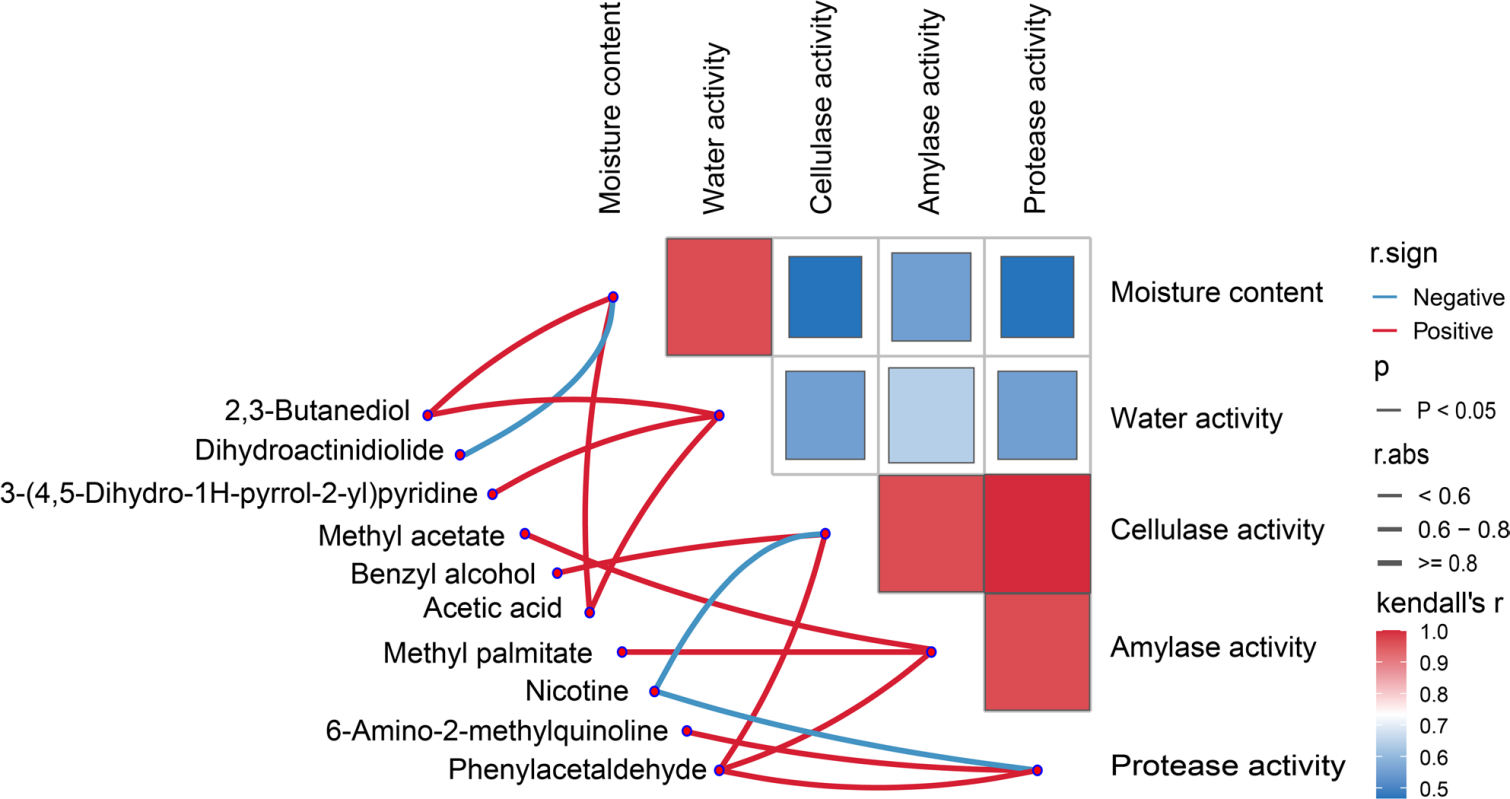
Correlation network analysis between water activity, enzymatic activities and VFCs

The regulation of key enzyme activities by water activity constitutes one of the core mechanisms governing the quality formation of CTLs (Li et al. 2018). Through hydrolyzing macromolecules, the enzyme system directly dictates the type and quantity of substrates indispensable for flavor synthesis, and its influence on VFCs is both systematic and phase-specific. It regulates the substrate supply chain and acts as a central hub within the multi-enzyme cooperative metabolic network. To elucidate the role of the enzyme system in modulating volatile compound synthesis during curing and fermentation, Kendall correlation analysis was performed on VFCs, water activity, and enzyme activity of cigars at each phase, as illustrated in Fig. 5. During the SDP, cellulase activity is notably elevated, degrading cell walls to release precursors like phenylalanine and directly facilitating benzyl alcohol synthesis. Upon entering the fermentation phase, protease activity reaches its peak, efficiently hydrolyzing proteins into free amino acids (e.g., phenylalanine, lysine) that generate 6-amino-2-methylquinoline through Maillard reactions and Strecker degradation. Meanwhile, nicotine content shows a significant negative correlation with cellulase and protease activities, and its degradation effectively enhances smoke mildness. Amylase activity rises synchronously at this stage, hydrolyzing starch into reducing sugars that act as substrates for Maillard reactions. This process also promotes the accumulation of aromatic esters (e.g., methyl acetate, methyl palmitate), thereby reinforcing fruity and fatty aroma notes (Banozic et al. 2020). Notably, the formation of phenylacetaldehyde—a core floral aroma constituent—exhibits a significant positive correlation with the activities of these three enzymes. Cellulase releases precursors, amylase supplies sugar moieties, and protease furnishes amino acids, thus systematically bridging macromolecular degradation and aroma synthesis (Banozic et al. 2020). 2,3-Butanediol exhibits a positive correlation with moisture content, indicating that a high-humidity environment facilitates the accumulation of sweet aroma precursors. Dihydroactinidiolide is negatively correlated with moisture content, reflecting that low moisture conditions (during the SDP) expedite carotenoid cleavage to produce floral and fruity aromas. 3-(4,5-Dihydro-1H-pyrrol-2-yl)pyridine correlates positively with water activity, demonstrating that the high-humidity environment in the early and middle fermentation phases fuels the formation of nitrogen-containing heterocyclic substances.

### Dynamic changes in microbial communities during air-curing and fermentation of CTLs

**Fig. 6:**
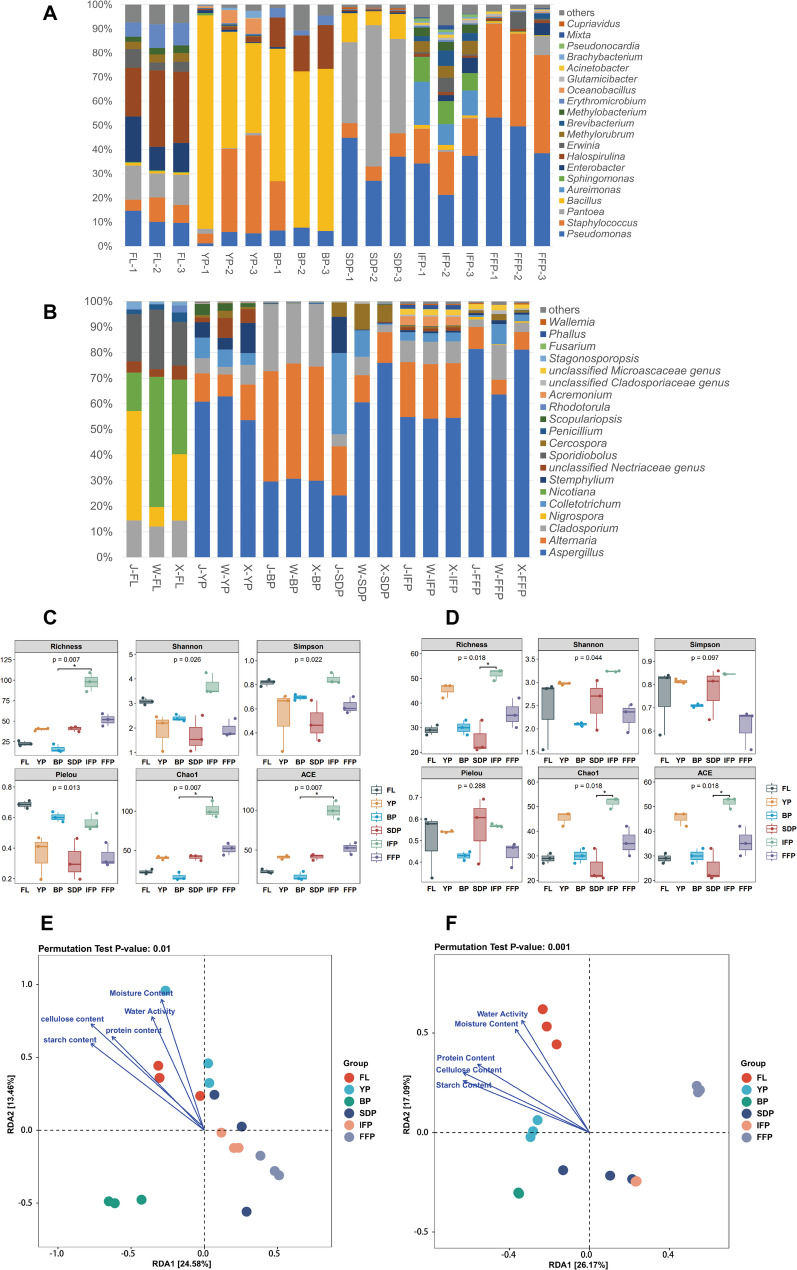
Microbial community composition and diversity across cigar tobacco processing phages. **A** Bacterial community composition at the genus level. **B** Fungal community composition at the genus level. **C** *α*-diversity indices of bacterial communities. **D** *α*-diversity indices of fungal communities. **E** RDA ordination of bacterial communities constrained by physicochemical parameters. **F** RDA ordination of fungal communities constrained by physicochemical parameters

Microorganisms improve cigar tobacco quality by breaking down macromolecular compounds and releasing aroma precursors, while simultaneously synthesizing key floral, fruity, and nutty aroma compounds during fermentation. In parallel, they reduce irritating constituents such as nicotine, thereby markedly enhancing the smoothness, harmony, and overall sensory quality of the CTLs (Zheng et al. 2022). Bacterial and fungal community analyses derived from 16S rRNA and ITS gene sequencing are presented in Fig. 6, At the fresh leaf phase, the bacterial community exhibited relatively high diversity but low overall abundance. Dominant genera mainly included *Halospirulina* (21.3%), *Enterobacter* (13.2%), *Pseudomonas* (11.8%), *Pantoea* (11.3%), and *Erythromicrobium* (8.9%), with *Halospirulina* and *Enterobacter* accounting for relatively higher relative abundances. During this same period, the fungal community was predominantly composed of taxa such as *Fusarium* (28.2%), *Nigrospora* (23.6%), *Cladosporium* (12.6%), and *Sporidiobolus* (15.6%). The high abundances of *Nigrospora* and *Fusarium* suggested that fungi at this phase were primarily derived from the endophytic and epiphytic microorganisms inherent to the tobacco plant, as well as residual microbial communities in the field environment, and had not yet experienced22 significant ecological selection imposed by air-curing or fermentation conditions. Upon transitioning to the YP, the microbial community structure underwent a marked shift. Within the bacterial community, *Bacillus* (66.8%) rapidly became the absolute dominant genus, with its abundance far surpassing that of other taxa; meanwhile, genera including *Staphylococcus* (15.6%), *Pseudomonas* (3.8%) were also significantly enriched. For fungi, total abundance increased substantially and the community structure was distinctly remodeled, with *Aspergillus* (58.5%) and *Alternaria* (10.2%) quickly establishing themselves as dominant genera. The sufficient moisture, optimal temperature and humidity conditions, and abundant available substrates during this phase provided a favorable environment for the rapid growth and metabolic activities of filamentous fungi (Mamy, Boateng et al. 2025). In the BP, overall microbial abundance declined significantly and the community structure tended to simplify, leaving only a few dominant genera—*Bacillus* (65.5%), *Halospirulina* (13.3%), and *Erythromicrobium* (3.3%) included. This indicated that with the continuous decrease in moisture and intensified oxidative stress, environmental selection pressure was markedly enhanced, permitting only highly adaptable microorganisms to persist. During this phase, the fungal community remained dominated by the core genera *Aspergillus* (29.9%) and *Alternaria* (43.5%), whose relative abundances stayed at high levels, while other field-derived or poorly adapted fungi decreased sharply. Consequently, the community structure shifted from a dispersed to a concentrated pattern.

During the SDP, the overall microbial load of CTLs remained low, while community composition exhibited relatively high stability. The bacterial community was dominated by *Pseudomonas* (33.5%), *Pantoea* (33.5%), and *Bacillus* (8.8%), with *Halospirulina* and *Enterobacter* still present at moderate proportions. This pattern indicates that under extremely low moisture conditions, overall microbial activity was strongly suppressed; however, dominant taxa with robust environmental adaptability and basal metabolic capacity were able to sustain a relatively stable community structure. The fungal community exhibited slight fluctuations in abundance at this phase but maintained structural stability overall. *Aspergillus* (52.5%) remained the dominant genus, while *Colletotrichum*, *Alternaria*, and *Cladosporium* persisted at appreciable levels. This suggests that although fungal proliferation was constrained by limited moisture and nutrient availability, stress-tolerant filamentous fungi retained the capacity to survive and sustain community stability.

Upon entering the IFP, microbial abundance increased markedly and community complexity was substantially enhanced. Within the bacterial community, *Pseudomonas* (29.5%) and *Staphylococcus* (14.7%) emerged as the dominant genera, while a wide range of functionally relevant taxa—including *Aureimonas* (11.5%), *Sphingomonas* (7.9%), *Methylorubrum* (4.4%), and *Methylobacterium* (3.4%)—proliferated extensively. This pattern reflects a typical enrichment of fermentation-associated microorganisms, indicating that microbial metabolism was highly active during this phase, with close links to the biosynthesis of aroma precursors and flavor compounds. By the FFP, total microbial abundance attained a relatively high level and community structure grew increasingly stable. *Pseudomonas* (46.4%) and *Staphylococcus* (40.4%) retained their dominant status, whereas the relative abundance of *Bacillus* declined markedly. Meanwhile, *Pantoea*, *Enterobacter*, and *Halospirulina* persisted at moderate levels, suggesting that a relatively balanced and functionally complementary microbial consortium had been established by the mature fermentation stage. Throughout the fermentation process, the fungal community also exhibited a pronounced increase in total abundance and evolved toward a highly concentrated structure. *Aspergillus* (74.1%) predominated overwhelmingly, accompanied by *Alternaria* (7.1%), *Cladosporium* (6.3%), and small proportions of stress-tolerant fungi including *Wallemia* and *Acremonium*. *Aspergillus* dominated the fungal community, corroborating previous findings on cigar tobacco fermentation (Zhang et al. 2023; Li et al. 2024). Species-level identification pinpointed *Aspergillus chevalieri* as the principal species—a non‑toxigenic agent involved in polysaccharide breakdown and volatile aroma production (Liu, Lai et al. 2026). The relatively stable fermentation environment during this phase further facilitated the selection and enrichment of functionally pivotal fungal taxa.

Throughout the entire curing–fermentation process of CTLs, variations in the species richness of bacterial and fungal communities were closely correlated with the gradual decline in water activity from 0.93 to 0.75, thereby reflecting the adaptive response of microbial communities to the moisture niche. At the bacterial community level, the fresh-leaf phase was dominated by field-derived microorganisms—including phyllosphere epiphytes and soil-borne taxa—with the consortium exhibiting limited functional potential. At the bacterial community level, the fresh-leaf phase was dominated by field-derived microorganisms—including phyllosphere epiphytes and soil-borne taxa—with the consortium exhibiting limited functional potential. Upon entering the YP, the abundance of metabolic bacteria such as *Bacillus* increased by more than fourfold, and community richness rose significantly relative to the fresh-leaf phase, marking this phase as a pivotal window for bacterial proliferation and functional reorganization. During the BP, as water activity plummeted, environmentally sensitive bacterial taxa were rapidly eliminated; community richness declined accordingly, leaving only a small number of stress-tolerant strains (e.g., *Bacillus licheniformis*) to dominate. In the SDP, bacterial richness dropped further to the lowest level observed throughout the entire process, with desiccation-tolerant taxa (e.g., *Sphingobacterium*, accounting for 67.8%) alone persisting. Upon entering the IFP, driven by constant temperature and humidity conditions, bacterial richness rebounded markedly, and the abundance of glycoside hydrolase genes associated with efficient decomposing taxa such as *Rhodococcus* increased threefold. By the FFP, bacterial richness decreased moderately yet remained significantly higher than that recorded in the late curing phase. At the fungal community level, the fresh-leaf phase was dominated by environment-derived taxa (e.g., *Fusarium*). During the YP, fungal richness increased markedly, whereas the Shannon index shifted distinctly—indicating that dominant fungal species such as *Scopulariopsis* and *Mycena* rapidly occupied ecological niches, with a concomitant reduction in community evenness. In the BP, fungal richness declined; by the SDP, however, it exhibited a slight recovery. Fungal richness reached its peak across the entire process during the IFP, with *Aspergillus* and *Penicillium* becoming significantly enriched. At the FFP, fungal richness declined, but the Shannon index remained at a relatively high level (3.24), as *Aspergillus* continued to dominate the process of aroma biosynthesis.

**Fig. 7:**
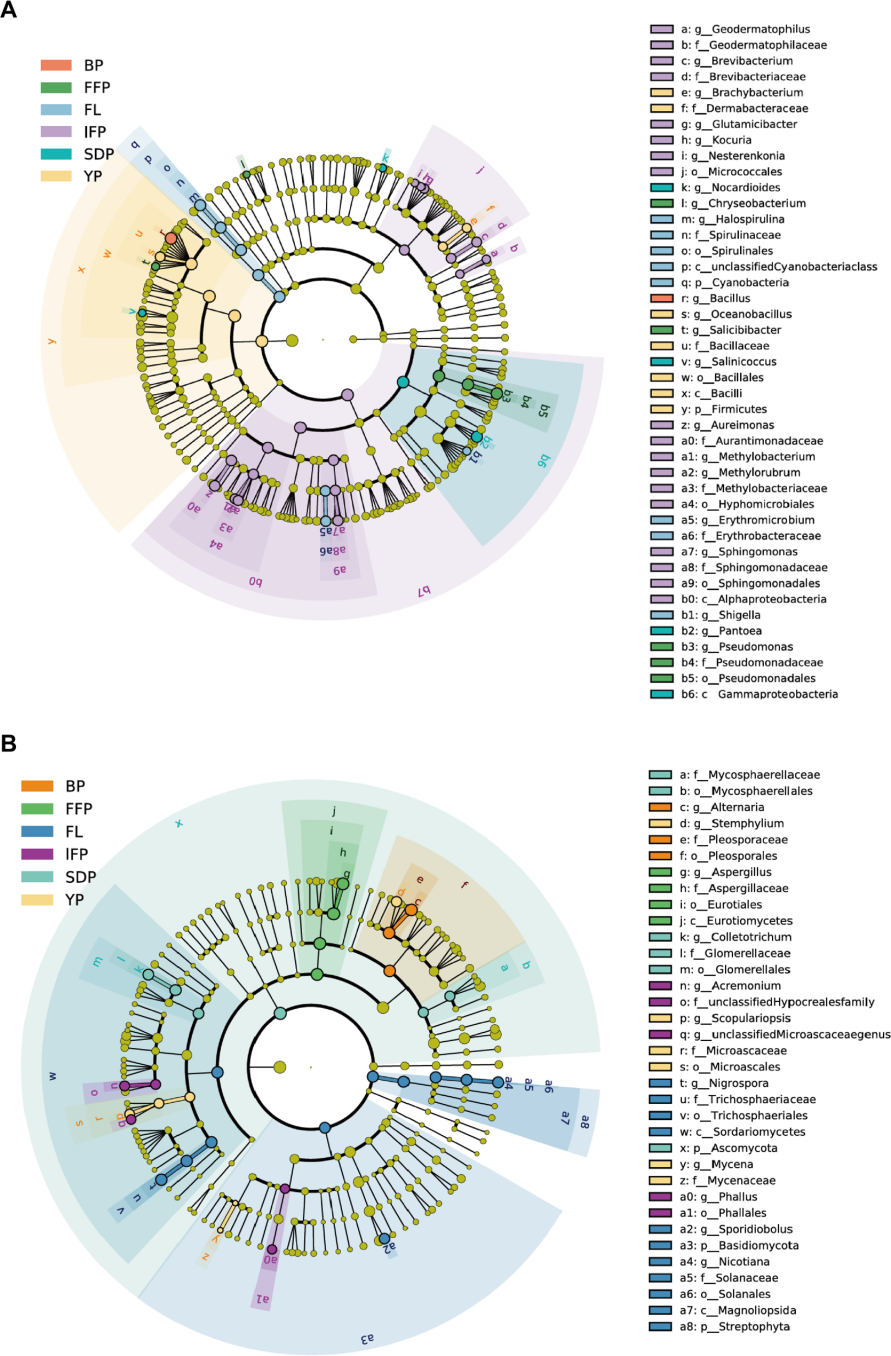
Characteristic microorganisms in cigars at different phases. **A** Phylogenetic distribution of fungal lineages in cigars. **B** Phylogenetic distribution of bacterial lineages in cigars

Results from the LEfSe analysis (LDA > 4, *p* < 0.05) demonstrated that throughout the entire cigar tobacco processing cycle, both bacterial and fungal communities underwent distinct function-oriented, stage-specific succession (as shown in Supplementary Fig. 4). Each processing phase corresponded to a unique, ecologically specialized biomarker taxon, reflecting the high adaptability of microbial communities to dynamic environmental variations and shifts in substrate composition (as shown in Fig. 7). During the fresh-leaf phase, the bacterial community was dominated by the order Spirulinales (family Spirulinaceae) within the photosynthetic autotrophic phylum Cyanobacteria, accompanied by the order Sphingomonadales (family Erythrobacteraceae) within the class Alphaproteobacteria. The fungal community was characterized by marker taxa belonging to the class Sordariomycetes and the order Trichosphaeriales. The microbial composition at this phase primarily reflected the primitive ecological traits of the raw material and had not yet undergone directional selection imposed by the processing procedure. Upon entering the YP, the bacterial community underwent substantial restructuring, with *Bacillus amyloliquefaciens* and *Bacillus subtilis* (family Bacillaceae) emerging as the dominant taxa. For the fungal community, the order Microascales (family Microascaceae, genus *Scopulariopsis*) and the family Mycenaceae (genus *Mycena*) were identified as the marker taxa. Upon entering the BP, the bacterial community became further concentrated in the genus *Bacillus*, dominated by the environmentally stress-tolerant species *Bacillus licheniformis* and *Bacillus pumilus*. In the fungal community, the order Pleosporales and its dominant genus *Alternaria* emerged as the predominant taxa. During the SDP, the bacterial community was characterized by the order Sphingobacteriales within the phylum Bacteroidetes as the marker taxon; the fungal community, in turn, exhibited significant enrichment of xerotolerant taxa belonging to the phylum Ascomycota, including the order Mycosphaerellales (class Dothideomycetes) and the order Glomerellales (class Sordariomycetes) along with its genus *Colletotrichum*.

Upon entering the IFP, the bacterial community was characterized by marker taxa from the phylum Proteobacteria, including the order Hyphomicrobiales (with *Rhodopseudomonas palustris* as the representative species) and the order Sphingomonadales (genus *Sphingomonas*). The fungal community was dominated by representative taxa such as the order Hypocreales (genus *Acremonium*) and the order Phallales (genus *Phallus*), a shift indicative of the transition in core microbial function from structural degradation to fermentation-associated metabolism. By the FFP, microbial functions had evolved toward high specialization: the bacterial community was centered on the genus *Pseudomonas*, with *Pseudomonas putida* and *Pseudomonas fluorescens* as the core species; eukaryotic microorganisms were absolutely dominated by *Aspergillus*, with the latter accounting for a relative abundance of 52.3%.

### Functional prediction of bacterial and fungal communities during air-curing and fermentation of CTLs

**Fig. 8:**
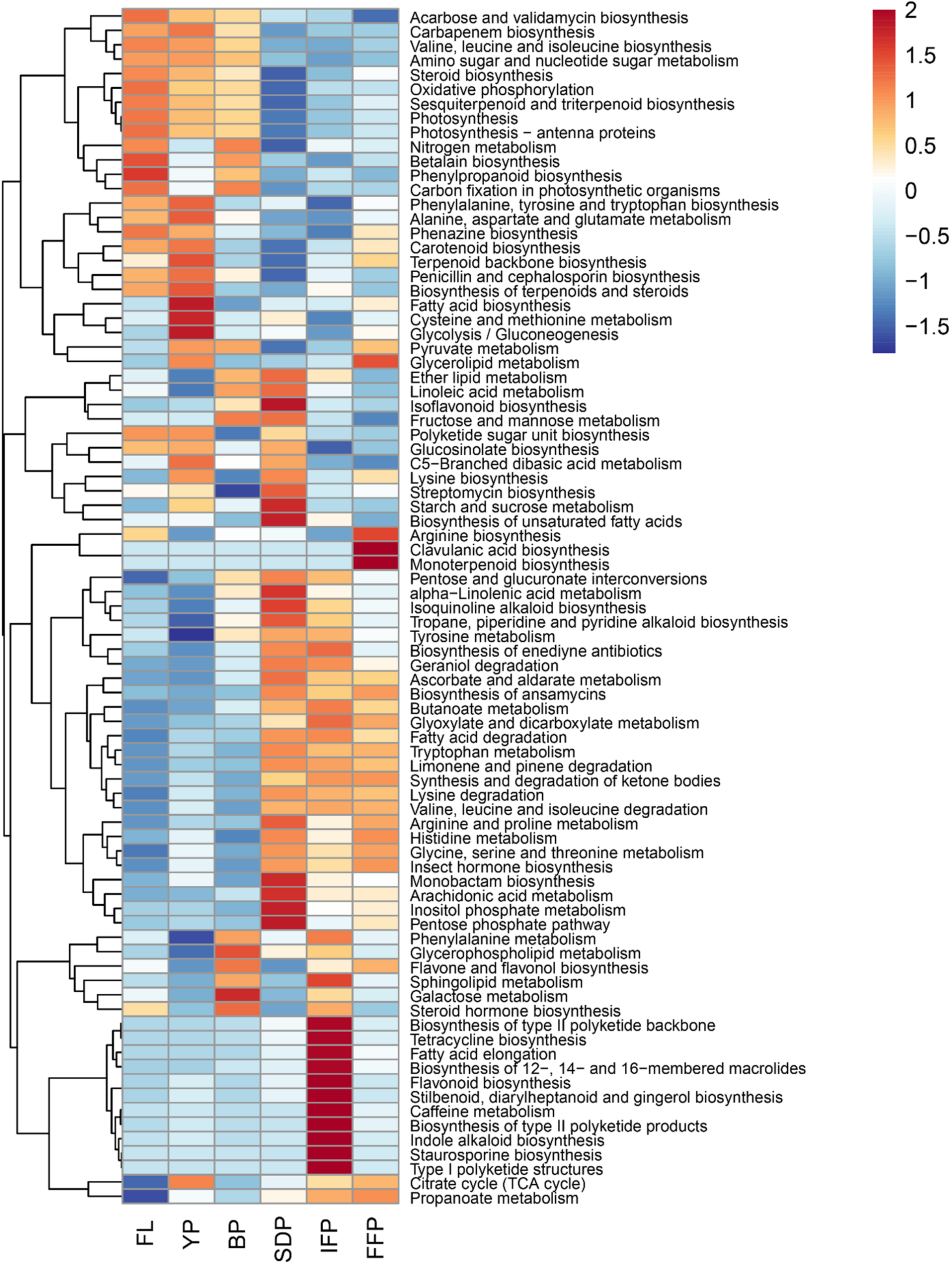
Heatmap of predicted KEGG Level 3 functional pathways

In this study, PICRUSt2 was utilized to perform functional potential annotation and analysis of the bacterial community at Level 2 and Level 3 during the curing and fermentation of CTLs, with the findings anchored in the metabolic pathway classification system of the Kyoto Encyclopedia of Genes and Genomes database. Based on the functional prediction results of KEGG Level 3 pathways (as shown in Fig. 8), the metabolic network during the fermentation phase is highly coordinated, with multiple functional modules (amino acid degradation, lipid oxidation, secondary metabolism) collectively driving the biosynthesis of flavor substances, thereby endowing cigars with complex yet harmonious sensory characteristics.

During the transition of cigar tobacco from the fresh-leaf phase through the curing phase to the fermentation phase,Valine, Leucine and Isoleucine Biosynthesis accounted for the highest proportion in amino acid metabolism across all stages. This indicates that branched-chain amino acid (BCAA) synthesis serves as a fundamental functional module of the bacterial community, supplying essential nitrogen sources for cell structure maintenance and primary metabolism. Meanwhile, pathways including Alanine, Aspartate and Glutamate Metabolism, Arginine Biosynthesis, and Phenylalanine, Tyrosine and Tryptophan Biosynthesis exhibited minimal fluctuations in abundance across different phases, confirming that basic nitrogen metabolism remains stable during curing and fermentation to fulfill the essential survival demands of the microbial community. In sharp contrast, amino acid degradation-related pathways (e.g., Lysine Degradation, Valine, Leucine and Isoleucine Degradation, Tryptophan Metabolism) increased significantly during the SDP and sustained a high level throughout the fermentation phase. Notably, the abundance of BCAA degradation pathways continued to escalate from the fresh-leaf phase to the FFP, suggesting that bacterial function transitions from a "synthetic maintenance type" to a "degradative transformation type" during air-curing and fermentation.

In terms of carbohydrate metabolism, Glycolysis/Gluconeogenesis, Pyruvate Metabolism, TCA Cycle, and Pentose Phosphate Pathway maintained high abundances across all phases, serving as the primary energy metabolism pathways. Upon entering the fermentation phase, Butanoate Metabolism and Propanoate Metabolism were continuously enhanced, indicating an increased bacterial involvement in short-chain fatty acid metabolism and fermentation-associated processes. Lipid metabolism function exhibited a significant upward trend from the SDP to the FFP. Fatty Acid Degradation reached a distinct high level during the SDP and sustained high abundance throughout the fermentation phase. Meanwhile, Biosynthesis of Unsaturated Fatty Acids, Linoleic Acid Metabolism were generally more abundant in the middle and late stages than in the early stages, reflecting a gradual enhancement of bacteria’s role in lipid transformation and the generation of oxidative derivatives. In particular, the linoleic acid is directly associated with the biosynthesis of key aroma components (e.g., nonanal, 2-pentylfuran), and the content of these aroma substances peaks at the final fermentation phase.

Regarding terpenoid and polyketide-related metabolism, Biosynthesis of Terpenoids and Steroids ranked as the secondary metabolism-associated module with the highest total abundance among all pathways, though its composition underwent significant phase-specific adjustments.Geraniol Degradation and Limonene and Pinene Degradation were significantly upregulated during the SDP (by 4.3-fold and 3.9-fold, respectively) and sustained high levels throughout the fermentation phase. The activation of these pathways facilitated the accumulation of carotenoid degradation products (e.g., *β*-damascenone, megastigmatrienone). Concurrently, Type II Polyketide Backbone/Products Biosynthesis was markedly enhanced during the fermentation phase (6.7-fold higher at the FFP than at the fresh-leaf phase), indicating that the secondary metabolic potential of bacteria is gradually activated during fermentation. Its products not only impart unique aromas but also exhibit antioxidant properties, which are beneficial for maintaining the long-term aging stability of CTLs. Notably, photosynthesis-related pathways (Photosynthesis, Photosynthesis–Antenna Proteins, Carbon Fixation in Photosynthetic Organisms) continuously declined from the fresh-leaf phase and remained at extremely low levels during fermentation.

**Fig. 9:**
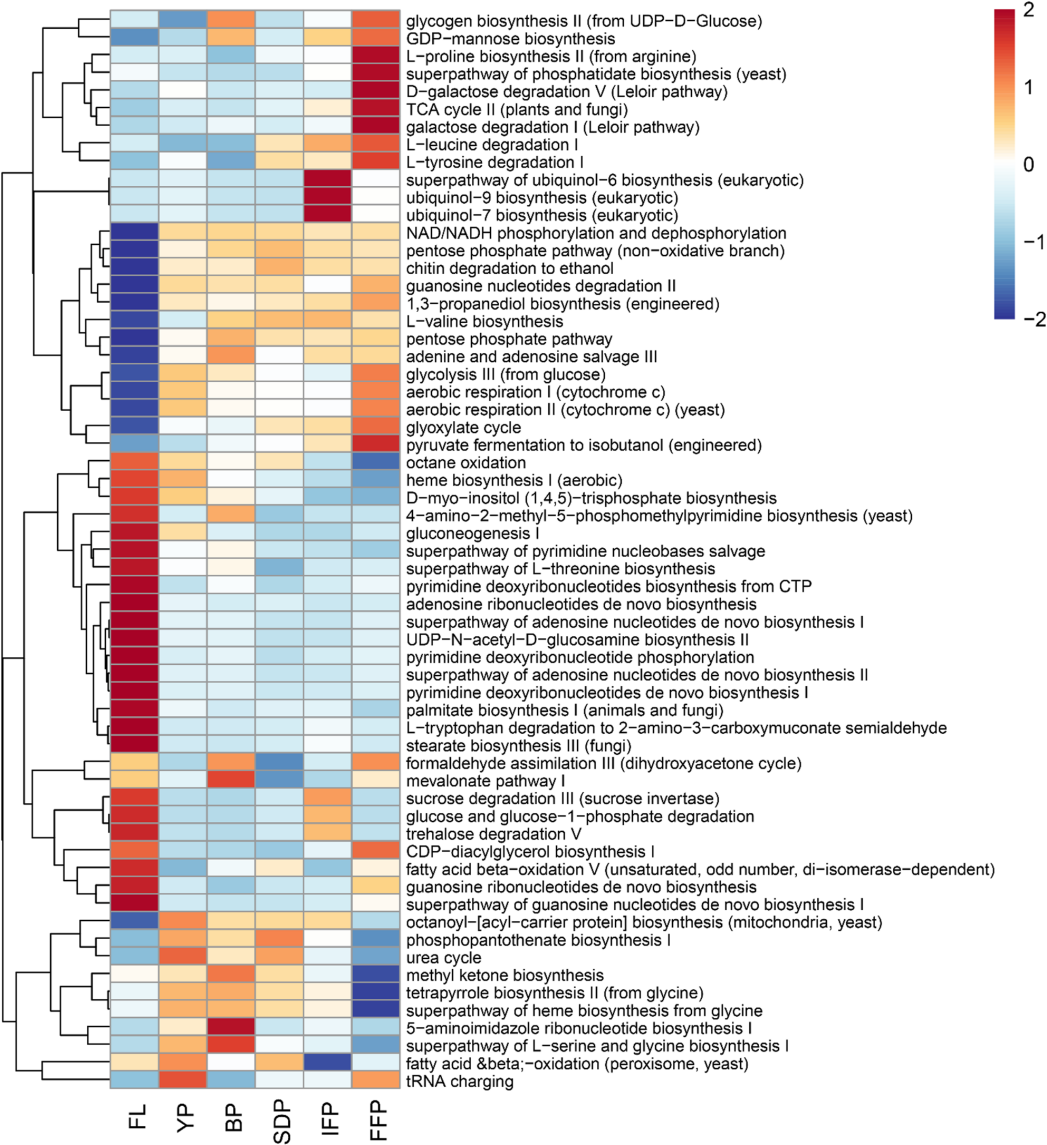
Predicted metabolic functions of the fungal community

Notably, KEGG functional prediction is generally not applied to fungal communities. This is not attributable to technical gaps, but rather constrained by multiple factors: insufficient coverage of reference genomes, poor correlation between ITS marker genes and functional genes, and inadequate representation of environmental fungi in the KEGG database—all of which compromise the reliability of prediction results. Fungal functional prediction was performed using MetaCyc, a database that exclusively curates experimentally validated pathways and offers broad coverage of fungal metabolism. Its EC-to-MetaCyc mapping is the standard output for ITS-based tools such as PICRUSt2, ensuring accurate and interpretable functional assignments (Karp and Caspi 2011; Caspi et al. 2016; Shrestha et al. 2022). Based on ITS amplicon sequencing data, the MetaCyc database was utilized to predict the functional groups of fungi across different processing phases of CTLs, as shown in Fig. 9.

Stage-specific variations were detected in the composition of fungal functional pathways across different processing phases, with pathways involved in basic substance metabolism and energy metabolism predominating overall. Throughout all stages, Glycolysis III, the Pentose Phosphate Pathway, TCA Cycle II, and Aerobic Respiration I/II maintained high relative abundances—indicating that the fungal community sustained robust basic metabolic activity during the entire processing process. Meanwhile, multiple pathways linked to amino acid metabolism, lipid metabolism, and nucleotide metabolism displayed distinct dynamic fluctuations across phases, reflecting the gradual shift of fungal community function from a "growth-maintenance type" to a "substance transformation and flavor precursor formation type."

During the fresh-leaf phase, fungal functional pathways were primarily oriented toward carbon source utilization and energy acquisition. The Glycolysis pathway, Pentose Phosphate Pathway (encompassing both oxidative and non-oxidative branches), and Glyoxylate Cycle all exhibited high relative abundances, indicating that the fungal community at this stage prioritized the rapid utilization of soluble carbohydrates and simple carbon sources. Meanwhile, pathways associated with de novo nucleotide biosynthesis (adenosine/guanosine nucleotides de novo biosynthesis), tRNA charging, and amino acid biosynthesis (e.g., L-serine, L-threonine, and L-valine biosynthesis) maintained stable levels, demonstrating that fungi in the fresh-leaf phase mainly performed functions related to cell proliferation and basic physiological activities. As CTLs transitioned into the yellowing and browning phases, the fungal functional lineage underwent marked adjustments. Relative to the fresh-leaf phase, pathways associated with the Glyoxylate Cycle, TCA Cycle, and aerobic respiration were substantially enhanced—indicating that fungi adapt to shifts in the internal physicochemical properties of CTLs by boosting respiratory metabolism efficiency under complex environmental stress. Notably, fungi from the genera *Aspergillus* and *Saccharomyces* exhibited a 5.8-fold upregulation in the expression of their species-specific amylase and cellulase genes, thereby accelerating the degradation of polysaccharides into fermentable sugars. Concurrently, the abundances of the Pentose Phosphate Pathway and pathways involved in NAD/NADH and NADP/NADPH interconversion were elevated, suggesting that the fungal community plays a crucial role in maintaining redox balance, supplying reducing power, and synthesizing metabolic precursors during this stage. For instance, fungal-mediated aromatic amino acid metabolism pathways (phenylalanine, tyrosine) were significantly upregulated, providing precursors for the biosynthesis of floral and fruity flavor compounds such as phenylethanol and benzaldehyde. These processes not only support the fungi’s own growth and reproduction but also establish the necessary metabolic foundation for the subsequent formation of flavor substances.

During the stem-drying and fermentation phases, fungal functional pathways further shifted their focus toward substance transformation and flavor precursor generation. The relative abundances of amino acid degradation-related pathways (e.g., L-leucine, L-tyrosine, L-tryptophan degradation) were significantly elevated, indicating that fungi engage in the in-depth decomposition of proteins and free amino acids at this stage, yielding volatile flavor precursors (e.g., methyl ketones, pyrazines). Furthermore, the fungus-specific ester synthesis pathway—with a 12.3-fold upregulation in the expression of alcohol acyltransferase genes—markedly facilitated the accumulation of fruity and waxy flavor components, such as phenethyl acetate and ethyl tetradecanoate. Lipid metabolism-related pathways—including fatty acid *β*-oxidation, phospholipid remodeling, and monoacylglycerol metabolism—along with methyl ketone biosynthesis and fatty acid oxidation pathways, were significantly enriched. These processes can directly or indirectly generate ketones, aldehydes, and fatty acid derivatives, serving as important precursors for the development of fatty, nutty, and aged flavors in CTLs (Yin et al. 2019).

### Interplay between the microbiome and metabolome during the air-curing and fermentation of CTLs

**Fig. 10:**
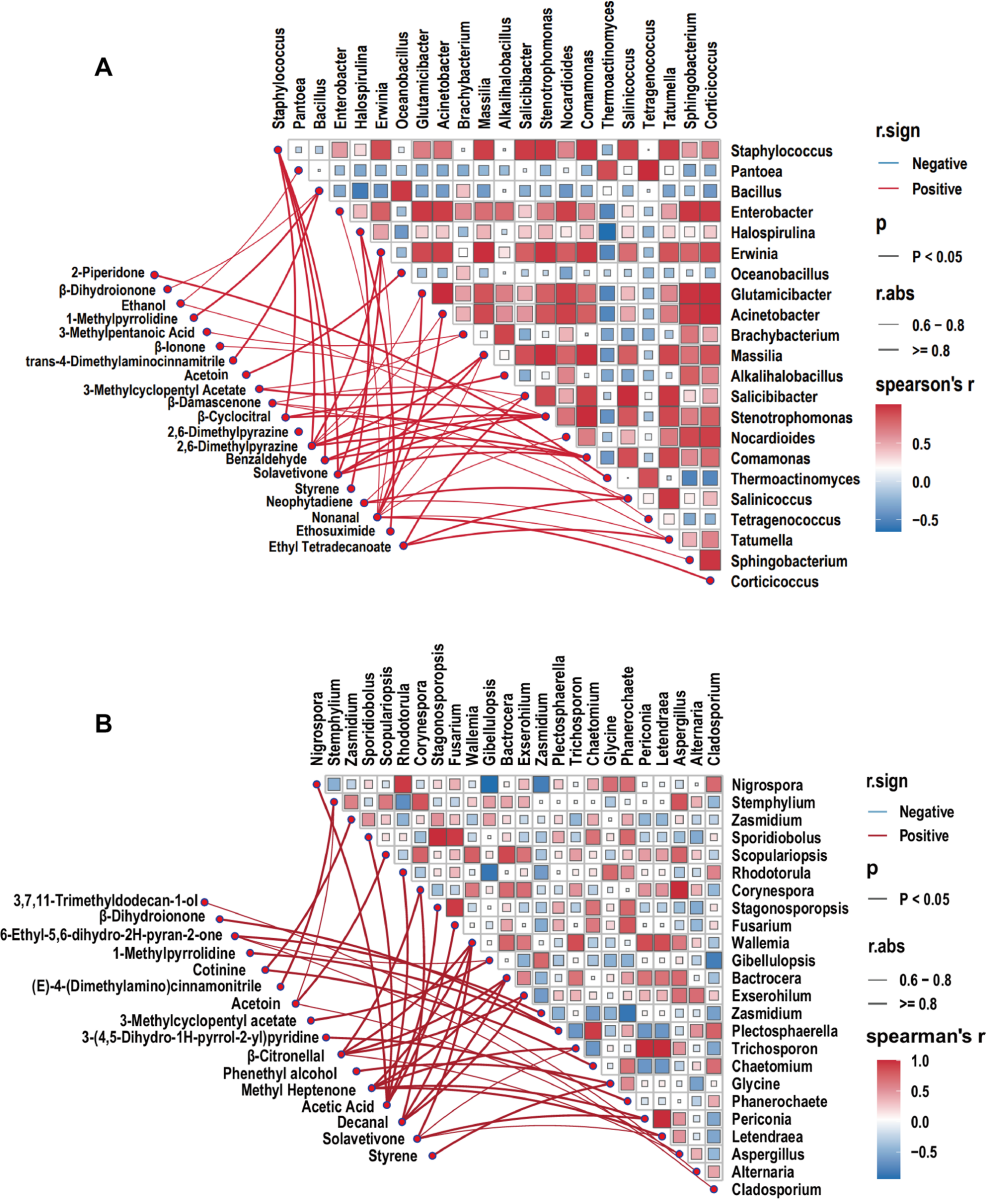
Correlation analysis between microorganisms and VFCs. **A** Spearman correlations between bacterial genera and VFCs. **B** Spearman correlations between fungal genera and VFCs

The formation of the cigar tobacco metabolome arises from the synergistic interplay of environmental factors, enzymatic reactions, and microbial community succession. Microorganisms not only initiate metabolic processes by driving macromolecular degradation and precursor release but also exert a key regulatory role in the generation, transformation, and homeostasis of aroma substances. The results of the correlation analysis between microbial community dynamics and metabolite variations during the air-curing and fermentation processes are shown in Fig. 10, phase-specific positive correlations between various dominant bacterial genera and specific volatile flavor substances. Notably, *Staphylococcus* showed significant positive correlations with *β*-cyclocitral, 2,6-dimethylpyrazine, benzaldehyde, and solavetivone—with the associated metabolites encompassing carotenoid cleavage products, pyrazine heterocyclic compounds, aromatic aldehydes, and sesquiterpenones. *Stenotrophomonas* also showed significant positive correlations with *β*-cyclocitral, *β*-ionone, 2,6-dimethylpyrazine, benzaldehyde, and solavetivone. The types of associated metabolites were highly consistent with those of *Staphylococcus*, but displayed a stronger preference for carotenoid cleavage products and compounds with high aroma intensity. *Bacillus* exhibited significant positive correlations with *β*-dihydroionone, 1-methylpyrrolidine, and trans-4-dimethylaminocinnamitrile, covering carotenoid degradation products, nitrogen-containing heterocycles, and aromatic nitrogenous compounds. Nonanal displayed positive correlations with multiple bacterial genera, such as *Sphingobacterium*, *Glutamicibacter*, *Acinetobacter*, *Halospirulina*, and *Erwinia*. This suggests that its formation is not driven by a single core functional bacterium, but rather linked to lipid oxidation processes co-mediated by diverse microbial taxa. Notably, *Oceanobacillus* showed a significant positive correlation with acetoin. As a typical compound imparting sweet and milky aromas, acetoin production is usually tightly associated with sugar metabolism pathways.

Multiple dominant fungal taxa displayed significant positive correlations with volatile flavor substances in CTLs, primarily encompassing carotenoid cleavage products, aldehydes, nitrogen-containing heterocyclic compounds, and aromatic metabolites. Among these, *Aspergillus* exhibited a significant positive correlation with *β*-citronellal, demonstrating a strong association between this fungal taxon and both the cleavage of carotenoid precursors and the accumulation of terpenoid aroma substances. Decanal is a pivotal aldehydic aroma compound, featuring an extremely low odor threshold, distinct citrus-fruity aromas, and extensive natural distribution (Lee et al. 2025). Decanal exhibited significant positive correlations with *Corynespora*, *Wallemia*, *Bactrocera*, and *Exserohilum*. Classified as a saturated aliphatic aldehyde, it boasts typical fruity aromas, with its formation typically linked to the oxidative degradation of fatty acids. These positive correlations between decanal and multiple microbial taxa demonstrate that the accumulation of this aliphatic aldehyde is characterized by distinct community synergy, rather than being dominated by a single fungal taxon. *Plectosphaerella* exhibited a significant positive correlation with *β*-dihydroionone. As a reduced derivative of *β*-ionone, *β*-dihydroionone features milder yet longer-lasting floral aromas, and this correlation highlights a close association between *Plectosphaerella* and the subsequent modification of carotenoid degradation products. Regarding nitrogen-containing heterocyclic compounds, Phanerochaete exhibited a significant positive correlation with 3-(4,5-dihydro-1H-pyrrol-2-yl)pyridine. Typically derived from nicotine metabolism or amino acid-related reactions, this compound’s significant association with Phanerochaete underscores a tight link between this fungal taxon and the biosynthesis of nitrogen-containing heterocyclic aroma substances. *Penicillium* is among the core functional fungal taxa most tightly linked to the accumulation of phenethyl alcohol and styrene, with its abundance dynamics showing a consistent trend with the aforementioned aromatic metabolites—highlighting a pivotal role in aromatic amino acid-related metabolic pathways.

## Discussion

The formation of cigar tobacco leaf quality is a multi-level synergistic process involving physical dehydration, enzymatic transformation, microbial metabolism, and chemical restructuring (Zhang et al. 2023; Gao et al. 2025a, b). This study identified water activity as the central regulatory factor during curing. This phase is characterized not only by a reduction in total moisture but also by a transition in water state (Pantongsuk et al. 2025). The rapid loss of free water suppresses undesirable spoilage microorganisms. During the SDP, the resulting low-moisture stress simplifies the microbial community structure, enriching only stress-tolerant taxa such as *Bacillus* and *Aspergillus*. Simultaneously, the retention of bound water helps maintain cell membrane integrity and preserves a crucial "reaction microenvironment" for subsequent enzymatic processes (Niu et al. 2024).

Throughout the air-curing process, cellulase persistently degraded cell wall polysaccharides, releasing aromatic precursors such as phenylalanine, which established the foundation for the formation of floral aroma compounds like benzyl alcohol (Ke et al. 2024). Concurrently, the elevated activity of amylase ensured a sustained supply of reducing sugars. This promoted esterification reactions, driving the formation of fruity- and fatty-scented esters, including methyl acetate and methyl palmitate. Protease played a dominant role in the efficient hydrolysis of proteins, significantly increasing the pool of free amino acids. This directly fueled the Maillard reaction and Strecker degradation, leading to the generation of key nitrogen-containing heterocyclic aroma substances, such as 6-amino-2-methylquinoline. Consequently, the metabolic focus shifted gradually from the degradation of structural components to the synthesis of flavor precursors. This transformation not only reduced irritating and undesirable constituents in the CTLs but also accumulated abundant utilizable carbon and nitrogen sources for subsequent microbial metabolism (Sun et al. 2011). Upon entering the SDP, as water activity declined to a low level of approximately 0.50, the activities of cellulase and amylase decreased significantly. In contrast, protease activity remained relatively stable. This indicates that under extremely low-moisture conditions, the degradation of polysaccharide substrates was inhibited, while the transformation of proteins and their degradation products remained ongoing. This phenomenon explains the notable increase in the diversity of heterocyclic compounds during the IFP. The sustained supply of amino acids—serving as direct precursors—enabled their conversion into various aroma-contributing heterocycles via multiple pathways, including the Maillard reaction, Strecker degradation, and microbial transformation. These shifts reflect a transition in the cigar flavor system from a phase of basic precursor accumulation to one of structural recombination, underscoring that the metabolic emphasis in the later curing phases had moved from structural carbon sources to the release and accumulation of nitrogen-containing precursors.

During the air-curing phase, overall microbial abundance remained relatively low, and community structure gradually simplified. In contrast, microbial richness and functional complexity recovered markedly during the fermentation phase, ultimately giving rise to a functionally specialized consortium dominated by *Pseudomonas* and *Aspergillus*. This transition reflects a clear shift from an “environment-input–driven” community toward a “function-oriented” fermentation microbiota. *Pseudomonas* exhibits high metabolic versatility and strong degradative capacity, secreting extracellular proteases and lipases that enable efficient breakdown of proteins and lipids. Numerous studies have also demonstrated its ability to degrade nicotine, thereby contributing to the reduction of irritancy in tobacco products (Zhong et al. 2010; Li et al. 2020). In addition, *Pseudomonas* can directly participate in aroma formation through active secondary metabolic pathways, such as polyketide biosynthesis, leading to the production of characteristic flavor compounds. *Aspergillus* is distinguished by a highly efficient extracellular enzyme system, including glycoside hydrolases, oxidoreductases, esterases, and proteases. This enzymatic repertoire enables the extensive degradation of plant cell walls, starch, lipids, and proteins, facilitating the release and transformation of key aroma precursors while simultaneously supplying accessible carbon sources to the broader microbial community. Notably, *Aspergillus* plays a central role in the cleavage and structural reorganization of terpenoid and carotenoid precursors via its specialized oxidative enzyme systems, directly generating key C_13_-norisoprenoid flavor compounds such as *β*-damascenone (rose-like aroma) and *β*-ionone (violet aroma) (Li et al. 2025). Furthermore, Aspergillus exhibits strong tolerance to low water activity, allowing it to persist and maintain metabolic activity during the SDP, thereby ensuring continuity in aroma precursor transformation (Huang et al. 2014; Dong et al. 2025).

During the FL phase, CTLs exhibited vigorous respiratory metabolism. The marked accumulation of low-molecular-weight aldehydes and alcohols, including isovaleraldehyde and 2-methylbutyraldehyde, together with acetic acid, reflects the maintenance of high tissue metabolic activity. With the onset of curing, however, the metabolic regime shifted substantially. Amino acid metabolism transitioned from biosynthetic pathways supporting cellular construction toward extensive catabolic pathways. Notably, the degradation of branched-chain amino acids (valine, leucine, and isoleucine) increased significantly from the SDP onward, indicating that CTLs began reallocating structural resources toward the accumulation of aroma precursors. Nicotine, the primary contributor to cigar harshness, underwent pronounced degradation as early as the YP, with its content decreasing by more than 60%. This process was accompanied by the transient formation of intermediates such as cotinine and 3-(4,5-dihydro-1H-pyrrol-2-yl)pyridine, confirming that nicotine was converted into less irritating derivatives through oxidative and demethylation pathways (Zhong et al. 2010). This transformation represents an initial “de-harshening” step in cigar quality formation. Further moisture reduction during the BP selectively enriched stress-tolerant microorganisms, primarily *Bacillus licheniformis* and *Bacillus pumilus*. Consistently, the activities of the geraniol degradation and limonene and pinene degradation pathways increased by 4.3-fold and 3.9-fold, respectively, thereby driving the directed biosynthesis of key aroma compounds such as *β*-damascenone and *β*-ionone.

In the SDP, even as macromolecular degradation nears completion, the initial formation of key aroma compounds and proto-aroma precursors commences, establishing the foundation for their rapid accumulation in the ensuing fermentation. The progressive decline in moisture during this phase suppresses overall microbial activity; however, high geraniol degradation activity is retained, predominantly driven by *Aspergillus*, thereby preserving terpenoid precursors for subsequent fermentation. The transient reduction in specific terpenoid compounds, including neophytadiene and dihydroactinidiolide, indicates their active utilization as substrates for further metabolic transformation. Notably, the SDP was characterized by the significant accumulation of 2-piperidone and ethyl phenylacetate, reflecting a fundamental metabolic shift from “substrate mobilization” to “quality shaping.” As a representative lactam, the pronounced increase in 2-piperidone suggests enhanced cyclization and structural rearrangement of degradation products derived from amino acids, alkaloids, and proteins under low water activity conditions (Banozic et al. 2020).This transformation reduces the potential irritancy associated with free amino acids and alkaloids and provides a molecular basis for the subsequent generation of heterocyclic aroma compounds during fermentation. Concurrently, the marked accumulation of ethyl phenylacetate highlights the early onset of aroma construction. This ester is typically formed via the esterification of aromatic substrates such as phenethyl alcohol and phenylacetic acid, a process dependent on the continuous release of aromatic amino acids—particularly phenylalanine—as well as sufficient pools of alcohols and organic acids (Li et al. 2018). Despite the relatively low moisture content, aromatic precursors and lipid-derived substrates accumulated during earlier curing phases supply adequate reactants to sustain ester biosynthesis.

By the FFP, total terpenoid content peaked, marked by a pronounced increase in neophytadiene and the collective emergence of floral-, fruity-, and woody-scented terpenoids such as *β*-damascenone, theaspirane, and valencene. Concurrently, the metabolic focus shifted toward the formation of various stable nitrogen‑containing heterocyclic compounds at low concentrations. Terpenoids and carotenoid degradation products—notably neophytadiene and *β*‑ionone—were significantly enriched in both diversity and abundance during late fermentation. These compounds impart complex aromatic nuances, such as floral and woody notes, which constitute the core of the cigar's perceived sophistication and roundedness. Furthermore, sustained esterification activity converted alcohols and acids into milder esters like ethyl nonanoate, effectively neutralizing pungent odors and harmonizing the overall flavor profile. Meanwhile, trace levels of heterocyclic compounds—including pyrazines and furans—contributed rich roasted and caramel-like notes, collectively underpinning the depth and layered complexity of the final cigar aroma.

Terpenoid degradation constitutes the core metabolic driver of typical cigar flavor formation (Qin et al. 2021). This study demonstrates that this process unfolds in distinct, sequential phases. During the transition from yellowing to browning, elevated activities of macromolecule-degrading enzymes facilitate the continuous accumulation of carotenoid-cleavage products, including dihydroactinidiolide, *β*-dihydroionone, and neophytadiene. Concurrently, moisture stress enriches a stress-tolerant functional consortium, prominently represented by *Aspergillus*. Within this microbial guild, the geraniol- and pinene-degradation pathways are upregulated by 4.3- and 3.9-fold, respectively, efficiently driving the targeted synthesis of key floral-fruity compounds such as *β*-damascenone. Upon entering the fermentation phase, as water activity recovers to approximately 0.75, *Aspergillus* establishes a functional synergy with *Pseudomonas*. The latter notably activates the type II polyketide backbone biosynthesis pathway, and together, they catalyze the production of high-impact aroma terpenoids, including solanone and *β*-ionone. By the final fermentation stage, the concentrations of various floral, fruity, and woody terpenoids—such as neophytadiene, theaspirane, and valencene—reach their peak. These dynamics underscore the tight coupling and synergistic interplay between microbial metabolism and non-enzymatic chemical degradation during flavor maturation, marking a qualitative shift in the aroma profile from a “precursor-release” stage to one of “advanced expression.”

Via microbe-metabolite correlation analysis, this study systematically clarifies the core functions and synergistic effects of key functional microorganisms in flavor compound synthesis during cigar fermentation. Staphylococcus is capable of directionally converting amino acids derived from protein degradation into key intermediates of the Maillard reaction and Strecker degradation, thereby robustly driving the biosynthesis of nitrogen-containing heterocyclic aroma compounds such as pyrazines (Wu et al. 2023). The significant positive correlation between *Staphylococcus* and metabolites including *β*-cyclocitral, 2,6-dimethylpyrazine, benzaldehyde, and solavetivone further demonstrates that its role extends beyond amino acid metabolism, extensively participating in multiple key flavor pathways such as carotenoid cleavage, aromatic aldehyde formation, and sesquiterpene transformation. This implies that *Staphylococcus* not only harbors active metabolic capacities for amino acids and reducing sugars, but may also modulate the local redox state to cooperate with other microorganisms in facilitating the conversion of fat-soluble precursors, thus promoting the simultaneous accumulation of various high-intensity aroma compounds (Pei et al. 2025). *Bacillus* harbors an efficient extracellular enzyme system, diverse secondary metabolite synthesis capacities, and a robust environmental stress response mechanism, thereby conferring functional dominance and ecological adaptability in complex fermentation systems (Zhao et al. 2025). This genus exhibits a significant positive correlation with *β*-dihydroionone and various nitrogen-containing compounds. By secreting diverse extracellular enzymes to continuously decompose structural substrates, *Bacillus* synergistically engages in carotenoid cleavage and the biosynthesis of nitrogen-containing heterocyclic compounds—an effect that not only reduces the content of irritating precursors but also markedly enhances the depth and complexity of cigar flavor.

The fungal community plays an irreplaceable role in the formation of high-grade aromas during the middle and late stages of fermentation. *Aspergillus* shows a significant correlation with *β*-citronellal. As a typical product of the carotenoid cleavage pathway, *β*-cyclocitral features fresh citrus and woody aroma notes, serving as a key component of terpenoid aromas in CTLs (Mosaferi et al. 2020). The core function of *Aspergillus* in the oxidation and structural modification of monoterpenoids enables the conversion of low-intensity terpenoid precursors into monoterpenoid aldehydes with lower sensory thresholds, thereby enhancing the fresh floral and fruity aromas of cigars. The positive correlation between decanal and *Corynespora*, *Wallemia*, and *Exserohilum* highlights distinct fungal synergy in aldehyde biosynthesis, endowing cigar flavor with a sense of maturity. *Plectosphaerella* is characterized by robust extracellular enzyme secretion capacity, active organic acid metabolism, abundant potential for secondary metabolite synthesis, and excellent tolerance to environmental stress (Zhao et al. 2025). Its significant association with *β*-dihydroionone suggests that this fungus may mediate the specific cleavage of carotenoid skeletons through its oxidase system. *Phanerochaete* exhibits a significant positive correlation with the nitrogen-containing heterocyclic compound 3-(4,5-dihydro-1H-pyrrol-2-yl)pyridine. Equipped with a potent oxidase system, *Phanerochaete* can efficiently degrade lignin—a trait that underscores the potential role of such strong oxidizing fungi in the in-depth rearrangement and cyclization of nitrogen-containing precursors. It imparts roasted, nutty, and smoky notes to cigars(Zhao et al. 2025).

Although this study mitigated the bias arising from regional specificity to a certain degree by collecting samples from three distinct producing regions within Hainan Province, it should be objectively acknowledged that the present work is limited in both spatial coverage and temporal scale, as the research was confined to a single province with a sampling period covering only one growing season, without involving broader geographical regions or successive planting years. The successional patterns of microbial communities and dynamic profiles of volatile metabolites in tobacco leaves revealed herein may vary depending on differences in climatic conditions, soil types, cultivation management regimes, and planting years across producing regions. Accordingly, the conclusions of this study are most directly applicable to cigar tobacco production systems with agronomic practices, ecological environments, and raw material characteristics comparable to those of Hainan. Future research should be expanded to multiple representative ecological producing regions to conduct multi-year and multi-site systematic validation, thereby providing a more systematic and robust scientific foundation for establishing scientific, precise, and region-specific fermentation process standards for cigar tobacco leaves.

## Conclusions

Water activity acts as the pivotal environmental regulator, directing biochemical transformations across successive processing stages. During the dehydration‑driven curing phase, cellulose, starch, and proteins are rapidly degraded, releasing essential flavor precursors—notably soluble sugars and free amino acids. Concurrent environmental selection enriches stress‑tolerant, functional microbial taxa, prominently represented by *Bacillus* and *Aspergillus*. As the process transitions into the fermentation phase, where moisture levels gradually recover, *Pseudomonas* and *Aspergillus* form a tightly coupled functional module. Through the coordinated activation of key metabolic pathways—such as polyketide biosynthesis and redox reactions—this microbial consortium synergistically drives the synthesis of defining flavor compounds, including terpenoids, esters, and nitrogen‑containing heterocycles.

## Supplementary Information

Below is the link to the electronic supplementary material.

Supplementary Material 1

Supplementary Material 2

## Data Availability

The datasets presented in this study can be found in online repositories. The names of the repository/repositories and accession number(s) can be found in the article/supplementary material.

## Abbreviations

CTLs: Cigar tobacco leaves
VFCs: Volatile flavor compounds
FL: Fresh leaf
YP: Yellowing phase
BP: Browning phase
SDP: Stem-drying phase
IFP: Initial fermentation phase
FFP: Final fermentation phase
