# Supplementary material for: Dynamic changes of volatile substances and their driving factors during curing and fermentation of Hainan cigar
Source: Bioresour Bioprocess. 2026 Jun 29;13(1):97. doi: 10.1186/s40643-026-01088-3 (PMC13310866; doi:10.1186/s40643-026-01088-3)
Supplement: Supplementary file 1 — Supplementary Material 1 [file 40643_2026_1088_MOESM1_ESM.docx]

Supplementary Material

# Supplementary Figures


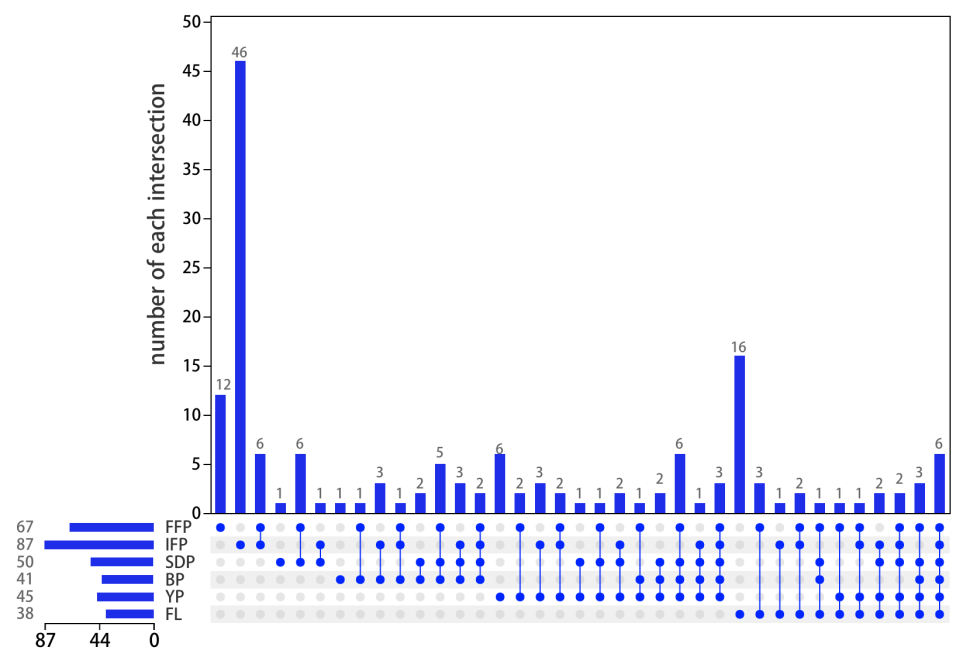


Supplementary Figure 1. Classification and composition of volatile compounds in cigar tobacco at different processing stages.


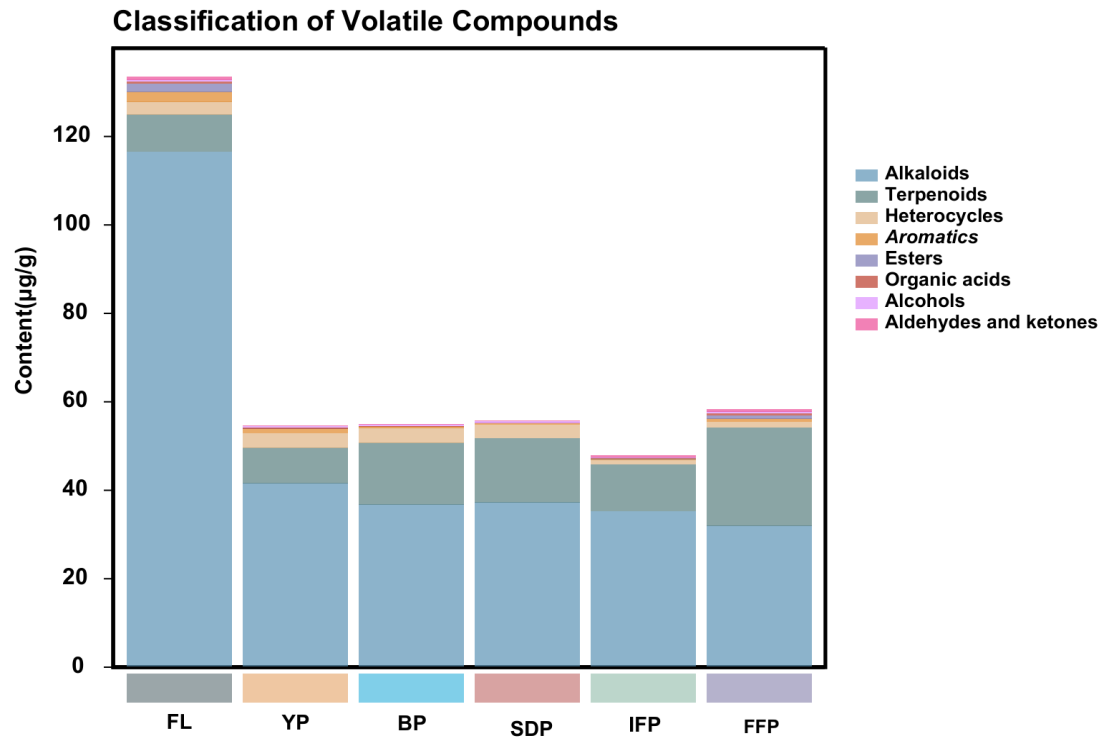


Supplementary Figure 2. Dynamic changes in the concentrations of various volatile compounds during the air-curing and fermentation of cigar tobacco.


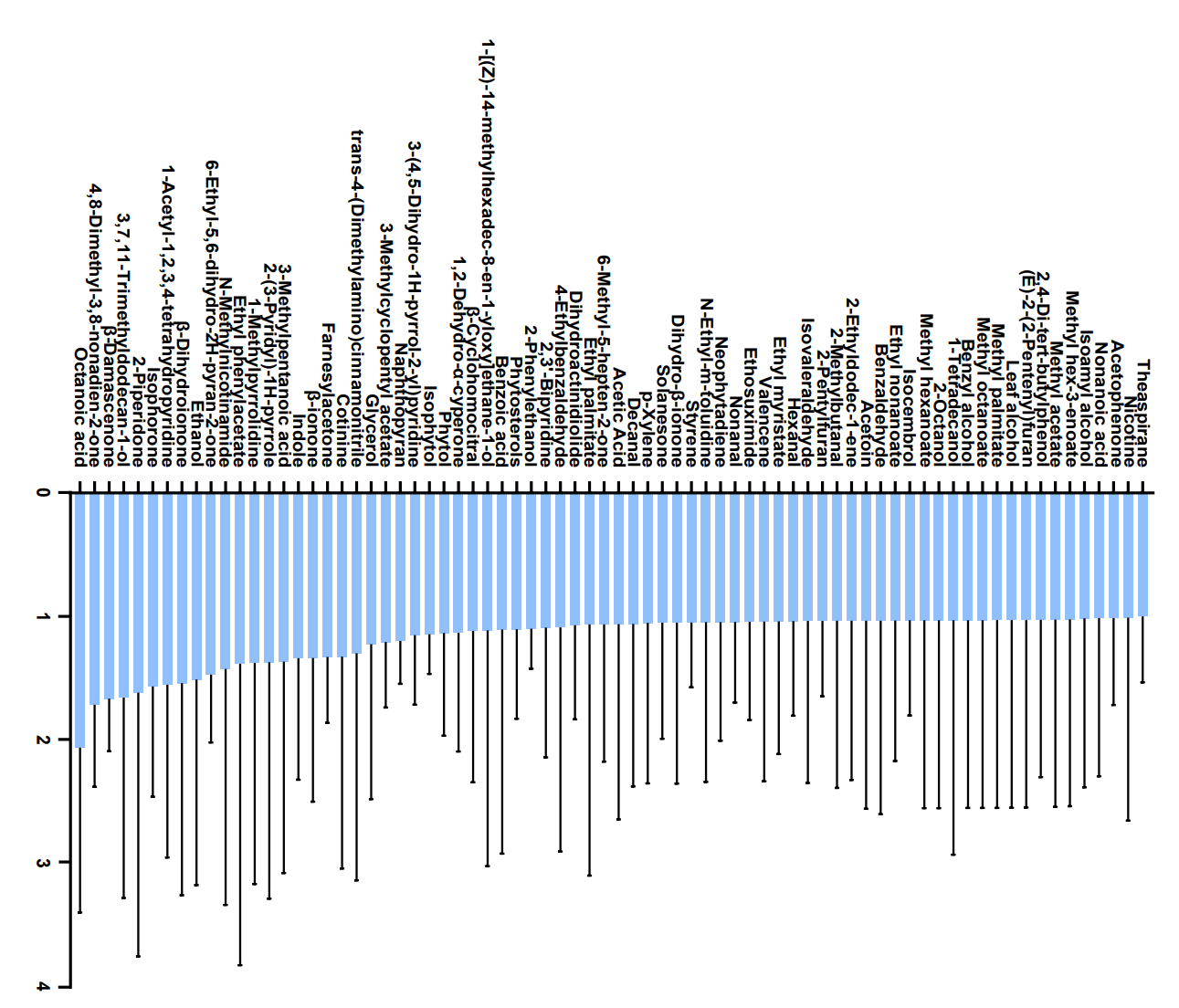


Supplementary Figure 3. Bar chart illustrating VIP values of volatile flavor compounds with VIP > 1 within the PLS-DA framework.


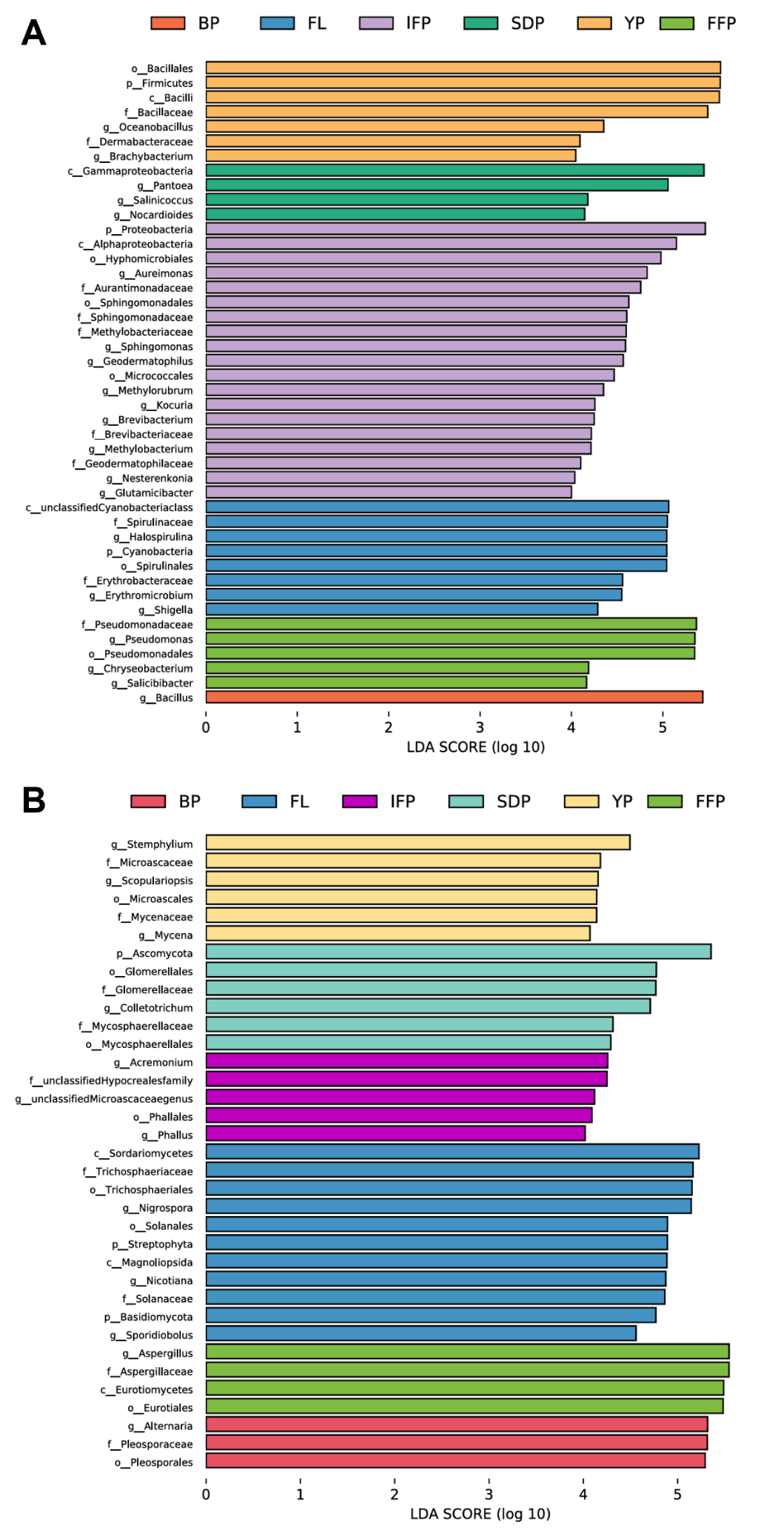


Supplementary Figure 4. Characteristic microorganisms in cigars at different phases. (A) Indicator fungal taxa with LDA > 3 in cigar microbial communities. (B) Indicator bacterial taxa with LDA > 3 in cigar microbial communities.
